# AI-driven discovery of antiretroviral drug bictegravir and etravirine as inhibitors against monkeypox and related poxviruses

**DOI:** 10.1038/s42003-025-09129-x

**Published:** 2025-12-02

**Authors:** Yining Wang, Atabey Ünlü, Xin Wang, Elif Çevrim, Dewy Mae Offermans, Myrthe P. Flesseman, Luca M. Zaeck, Liping Wu, Marcel J. C. Bijvelds, Nadia A. Sam-Agudu, Rory D. de Vries, Karine Raymond, Pengfei Li, Abdurrahman Olğaç, Wenshi Wang, Tunca Doğan, Qiuwei Pan

**Affiliations:** 1 School of Life Sciences and Biopharmaceutical Sciences, Shenyang Pharmaceutical University, Shenyang, China; 2 Department of Gastroenterology and Hepatology, Erasmus MC-University Medical Center, Rotterdam, The Netherlands; 3 Biological Data Science Lab, Department of Computer Engineering, Hacettepe University, Ankara, Turkey; 4 Department of Bioinformatics, Graduate School of Health Sciences, Hacettepe University, Ankara, Turkey; 5 Department of Anatomy and Embryology, Leiden University Medical Center, Leiden, The Netherlands; 6 The Novo Nordisk Foundation Center for Stem Cell Medicine (reNEW), Leiden University Medical Center, Leiden, The Netherlands; 7 Department of Viroscience, Erasmus MC-University Medical Center, Rotterdam, The Netherlands; 8 International Research Center of Excellence, Institute of Human Virology Nigeria, Abuja, Nigeria; 9 Department of Pediatrics and Child Health, School of Medical Sciences, University of Cape Coast, Cape Coast, Ghana; 10 Global Pediatrics Program and Division of Infectious Diseases, Department of Pediatrics, University of Minnesota Medical School, Minneapolis, MN USA; 11 University of Grenoble Alpes, CEA, Inserm, IRIG, UA13 BGE, Biomics, Grenoble, France; 12 Department of Pharmaceutical Chemistry, Faculty of Pharmacy, Gazi University, Ankara, Turkey; 13 Department of Pathogen Biology and Immunology, Jiangsu Key Laboratory of Immunity and Metabolism, Jiangsu International Laboratory of Immunity and Metabolism, Xuzhou Medical University, Xuzhou, China; 14 Department of Health Informatics, Institute of Informatics, Hacettepe University, Ankara, Turkey

**Keywords:** Molecular medicine, Pharmaceutics

## Abstract

Monkeypox virus (MPXV) caused the 2022–2023 global mpox and the concurrent outbreaks in Africa, disproportionately affecting immunocompromised individuals such as people living with HIV. With no approved treatment available, we developed a robust artificial intelligence (AI) pipeline for discovering broad-spectrum poxvirus inhibitors that target the viral DNA polymerases. Among the identified leading candidates, we found that the clinically used antiretroviral drugs bictegravir and etravirine potently inhibit MPXV clade Ia, Ib and IIb infections in human intestinal and skin organoids. The broad anti-poxvirus activities of bictegravir and etravirine were further demonstrated against infections of other *Orthopoxviruses* such as vaccinia virus and cowpox virus. These findings support the repurposing of bictegravir and etravirine for treating mpox, especially for patients co-infected with HIV, warranting follow-up clinical investigation. The established AI pipeline and our antiviral drug discovery strategies bear major implications for responding to the ongoing mpox emergency and preparing for future poxvirus epidemics.

## Abstract (web summary)

AI-driven discovery of the antiretroviral drugs bictegravir and etravirine as potent inhibitors against monkeypox and related poxviruses infection. The effects were experimentally validated in human organoids infected with poxviruses.

## Introduction

In May 2022, while the COVID-19 pandemic was still evolving, the world faced another public health threat—a global outbreak of mpox (formerly known as monkeypox). Europe was the initial epicenter, but the disease rapidly spread to over 100 countries across the globe^1^. In July 2022, the World Health Organization (WHO) declared a public health emergency of international concern (PHEIC), but the emergency status was ended 10 months later as the number of reported cases declined. In 2023, in response to the emergence of a new major outbreak in the Democratic Republic of the Congo (DRC), and the upsurge in cases and further spread to other African countries, the WHO declared another mpox PHEIC in August 2024^2^.

Skin lesions are the most classical symptom of mpox, but monkeypox virus (MPXV) infection can cause a wide range of systemic manifestations including neurological injury, diarrhea, proctitis, liver injury, acute kidney injury, respiratory complications, and even death^3,4^. MPXV is a zoonotic pathogen belonging to the *Orthopoxvirus* genus of the Poxviridae family, which are large, enveloped, DNA viruses. Genetically, MPXV is classified into clade I with subclades Ia and Ib, and clade II with subclades IIa and IIb. Clade IIb MPXV caused the 2022–2023 global outbreak^1^. However, since early 2025, Sierra Leone has experienced its largest recorded mpox outbreak to date, marking the first major epidemic in Africa caused by the clade IIb strain^5^. Clade Ia and Ib are causing the ongoing outbreaks in Central and East Africa^6,7^. Clade I MPXV is thought to be more pathogenic than clade II strains, historically resulting in higher case fatality rates^8^. Other members of the *Orthopoxvirus* genus include now extinct variola virus (the cause of the deadly smallpox), the vaccinia virus (used in the smallpox vaccine), as well as the cowpox, rabbitpox and camelpox viruses. Occupational spillover infections from animal hosts to humans have been reported for cowpox, but rarely for rabbitpox and camelpox viruses^9^.

Currently, there is no approved treatment for mpox. The antiviral drug tecovirimat, approved for treating smallpox, has recently been authorized under exceptional circumstances by European Medicines Agency, and is administered under an expanded access protocol held by Centers for Disease Control and Prevention (CDC) of the United States for treating mpox^10^. Astonishingly, two recent randomized, placebo-controlled trials showed no clinical benefit of oral tecovirimat treatment for patients infected with clade I^11^ or clade II (https://www.natap.org/2025/CROI/croi_127.htm) MPXV strains. Furthermore, the detection of tecovirimat-resistant MPXV strains in treated patients has raised further concerns^12,13^. Cidofovir and its prodrug brincidofovir are DNA polymerase inhibitors that have been shown to inhibit the replication of many DNA viruses. These drugs have been occasionally prescribed as off-label treatment for mpox, but the efficacy remains unknown^14^. A randomized, double-blind, placebo-controlled trial is currently underway in Africa mainly in the DRC to assess the safety and efficacy of brincidofovir in treating mpox (https://africacdc.org/news-item/enrollment-starts-in-africa-cdc-led-mpox-therapeutic-study-mosa/). The lack of safe and effective mpox treatments has been a prevailing challenge, especially for disproportionately affected children in African countries as well as immunocompromised people and pregnant women, globally^15,16^.

To expedite research and develop medical countermeasures to respond to epidemics and pandemics, the WHO has implemented a viral-family based pathogen prioritization strategy. The WHO scored the Poxviridae family as high risk for causing a future PHEIC, and MPXV was listed as a prioritized and prototype pathogen^17^. This study aims to develop an innovative pipelines for discovering, testing and validating drug candidates possessing broad-spectrum antiviral activity against viral pathogens, albeit focusing on MPXV and the related poxviruses. To peruse this endeavor, we here integrate artificial intelligence (AI) and human intestinal or skin organoid-based infection models to prioritize the repurposing of medications already in clinical use, responding to the urgent medical needs of combating the ongoing mpox outbreak.

## Results

### Key features of an AI-based pipeline for broad-spectrum poxvirus inhibitor discovery

**Fig. 1:**
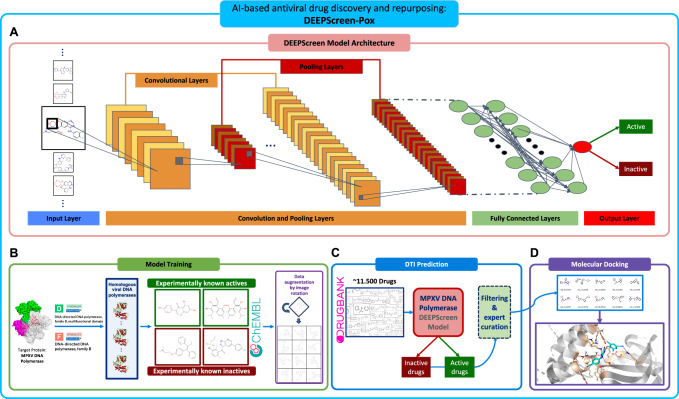
Schematic representation of the AI-based pipeline for anti-poxvirus drug discovery and repurposing. **A** The model architecture of the DEEPScreen system: The target-specific models of DEEPScreen take 2-dimensional image representations of compounds in the training dataset as input and process them with CNNs composed of subsequent convolution and pooling layers. The molecular representation learned by the convolutional layers is then transformed into binary prediction (as “active” or “inactive” considering its bioactivity against the target protein of interest) using multiple fully connected layers. **B** The training dataset of a target-based DEEPScreen model is composed of experimentally known active ligands and inactive molecules of the selected target (or highly similar homologs if there are no available experimentally identified ligands for the selected target, which is the case in this study), obtained from the ChEMBL database. The input compound images are augmented with 10-degree rotations, creating 36 images per molecule, yielding rotation invariance. In this study, we trained a prediction model for the MPXV DNA polymerase protein by curating a training dataset of 251 small molecules with active/inactive cutoff value of pChEMBL: 5.8 (IC50/EC50/AC50: ~1.5 μM). **C** The trained model is utilized to predict the activity of ~11.500 drug entries from DrugBank to find potential inhibitors of MPXV DNA polymerase. **D** Molecules selected from active predictions are used in the molecular docking analysis to finalize the drug candidates for further experimental validation.

AI-driven approaches (e.g., machine/deep learning) are widely employed in drug discovery^18,19^. Here, we utilized our deep learning-based drug-target interaction (DTI) prediction framework, DEEPScreen^20^, to develop a robust pipeline for anti-poxvirus drug discovery, named DEEPScreen-Pox. DEEPScreen outperforms state-of-the-art DTI prediction models on multiple benchmarks (e.g., on the ChEMBL temporal split dataset, DEEPScreen scored an average MCC = 0.45, 36% higher vs. the next deep model; on the MUV benchmark, DEEPScreen scored an average MCC = 0.81, 88% higher compared to the best performing baseline model), as reported in our previous work^20^. This model predicts DTIs by analyzing 2D compound images using convolutional neural networks (CNNs) to learn molecular features and classify compounds as active or inactive against a given target protein (Fig. 1A).

Viral DNA polymerases are responsible for the replication of DNA viruses. They are well-conserved, at least between members of the same viral family. To respond to the current mpox outbreak, but also to prepare for future poxvirus epidemics, we designed our pipeline to broadly target the well-conserved viral DNA polymerases of the Poxviridae family, albeit focusing on MPXV (Fig. 1B).

We trained the DEEPScreen-Pox model by curating a training dataset composed of experimental bioactivity data for the ligand binding region on the “DNA polymerase family B domain” (InterPro: IPR006134) of highly similar viral DNA polymerases. Since no experimental bioactivity data are available for the MPXV DNA polymerase at the time of developing this pipeline, we included two polymerases from other prototypic *Orthopoxvirus*, vaccinia virus (VACV) and variola virus (VARV), together with other DNA viruses, which have experimental bioactivity data (Supplementary Table 1). This resulted in a training dataset of 251 small molecules, and the bioactivity data distribution of this dataset is shown in Supplementary Fig. 1. The finalized dataset was divided into training, validation and test folds using a scaffold-based split (Fig. 1C). Each molecule’s SMILES notation was converted into 300 × 300 pixel images, and data augmentation was applied by rotating each image in 10-degree increments, generating 36 images per molecule and 9036 data points in total. The DEEPScreen-Pox model was optimized using a grid-based hyperparameter search. The best model, determined by the highest validation Matthew’s Correlation Coefficient (MCC) score, used a batch size of 64, a dropout rate of 0.2, and a learning rate of 0.00001 and was trained for 100 epochs. This model achieved high-performance results on the hold-out test dataset (i.e., F1-score: 0.858, Accuracy: 0.898, and MCC: 0.784), indicating its robustness (Supplementary Fig. 2). The DEEPScreen-Pox model is openly available together with its datasets at https://github.com/HUBioDataLab/DEEPScreen2. Although functional bioactivity data specific to MPXV were not available for model training—so only data from other *Orthopoxviruses* could be used—we still observed high predictive performance. This appears to result from the high conservation of key *Orthopoxvirus* proteins^21^. A similar transfer strategy has been employed in previous studies to prioritize MPXV inhibitors^22^.

### Sequence-based, structural and functional correspondence between *Orthopoxvirus* DNA polymerases

To assess conservation between MPXV and other viral proteins included in our DEEPScreen model training dataset, we analyzed sequence, structural, and functional similarities among the DNA polymerases of MPXV (UniProtKB: A0A7H0DN44), VACV (UniProtKB: P20509), and VARV (UniProtKB: P0DOO5). Pairwise global sequence alignment analysis revealed a high degree of conservation, with MPXV sharing 98.4% identity with VACV and 97.4% with VARV at the full-length protein level. Within the DNA polymerase family B domain (InterPro: IPR006134), which contains the binding pocket, sequence identity further increased to 99.2% with VACV and 98.2% with VARV, highlighting conservation of residues essential for catalytic activity (Supplementary Data 1–4).

For the structural similarity analysis, protein–ligand complex models were subsequently generated using the AF3 model^23^ for MPXV, VACV, and VARV DNA polymerases bound to native ligand from the reference structure (PDB code “8HG1”, native ligand: TTP). The AF3’s native metrics indicated that TTP ligand-bound complexes yielded high-confidence structures for MPXV, VACV, and VARV polymerases, with iPTM values of 0.89–0.91, pTM scores of 0.91–0.92, and mean pLDDT values of 88.1–88.7, supporting their reliable use in subsequent analyses (Supplementary Table 2). Structural similarity analysis of the predicted complexes demonstrated that MPXV and VARV DNA polymerases were the most similar, with a backbone RMSD of 0.345 Å, followed by VACV–VARV (0.349 Å) and MPXV–VACV (0.394 Å). Binding site comparisons, defined as residues within 5 Å of the ligand, revealed even higher similarity values: MPXV–VARV (0.060 Å), MPXV–VACV (0.076 Å), and VACV–VARV (0.086 Å), indicating strong conservation between the given proteins, especially considering binding regions. AF3 prediction files are provided in Supplementary Data 5.

Analysis of Gene Ontology (GO) annotations provided insights into the functional similarity of the DNA polymerases. MPXV DNA polymerase is annotated with 12 GO terms (Supplementary Table 3), and all of these terms are shared with VACV DNA polymerase, which has one additional GO term annotation (similarity score: 0.96). Similarly, all 12 annotated GO terms of MPXV are shared with VARV, which have 2 additional annotations (similarity score: 0.92). Please see Supplementary Table 3 for detailed information about the GO annotations of these proteins. Collectively, these results demonstrate that VACV and VARV DNA polymerases closely recapitulate both the structural and functional characteristics of MPXV DNA polymerase, particularly at the ligand-binding site, supporting their use as reliable surrogates in ligand interaction prediction.

### Anti-poxvirus drug discovery and repurposing by DEEPScreen-Pox

To discover drug candidates that can be expeditiously proceeded into clinical testing through drug repurposing, this study prioritized clinically approved and investigational small molecule drugs curated in DrugBank^24^. We screened all entries (i.e., ~11,500 small molecules) by DEEPScreen-Pox followed by a confidence score-based filtering (≥70%) and molecular size-based elimination, together with expert curation in parallel (Fig. 1C). Ultimately, we identified 50 potential candidates with broad-spectrum inhibitory activity on the poxvirus DNA polymerases (Fig. 1D and Supplementary Table 4).

**Fig. 2:**
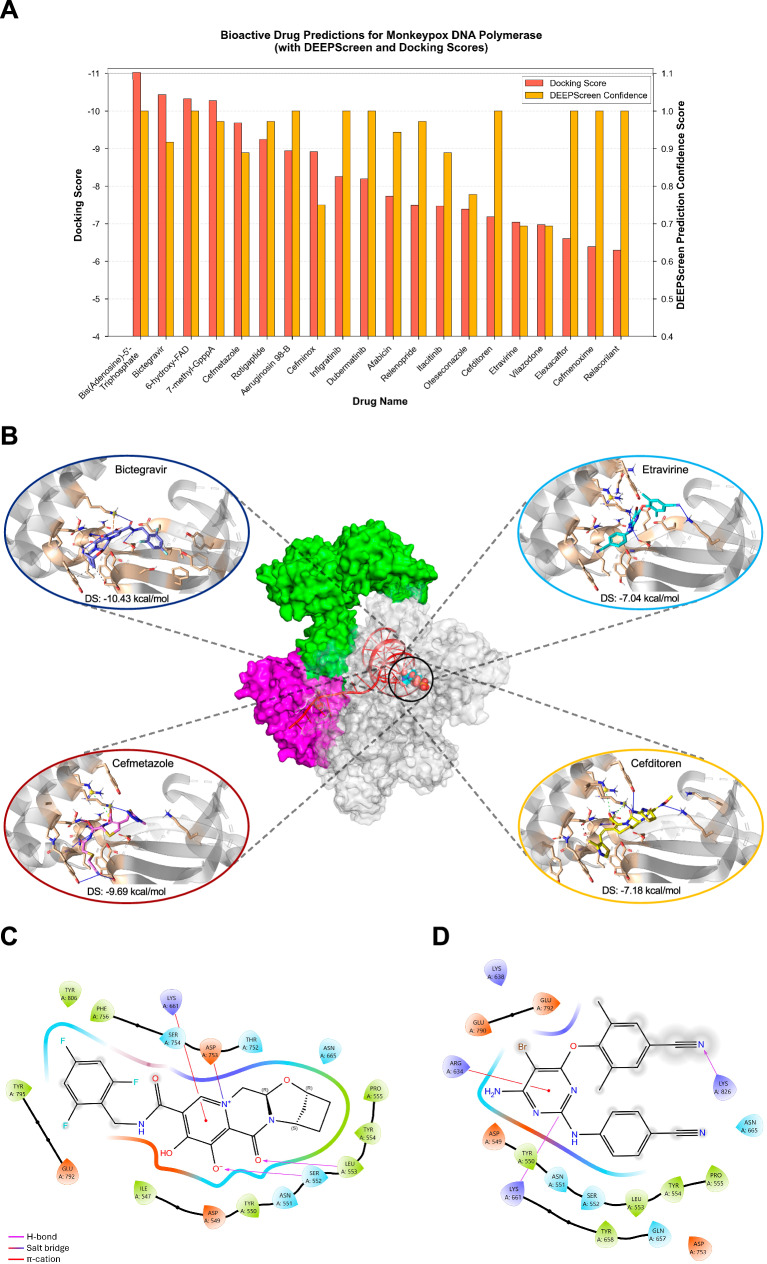
Computational drug repurposing analyses. **A** Bar graph depicting molecular docking and DEEPScreen prediction confidence scores for the top 20 drug candidates, ranked by their docking performance. **B** The output of the docking analysis of the selected four drugs: bictegravir, cefmetazole, cefditoren and etravirine, visualized in 3 dimensions. The interactions are displayed together with the residues in the active site of MPXV DNA polymerase holoenzyme (PDB code “8HG1“). The residues within a 4 Å cut-off, measured from the ligands, are depicted in wheat color and represented as sticks, surrounded by the secondary structural elements in the binding pocket. The complete structure of the MPXV holoenzyme (in the center): It contains DNA polymerase F8 (gray), processive cofactors A22 (green) and E4 (magenta), the primer-template DNA (red), and thymidine-5′-triphosphate (TTP) substrate (in the circle). Bond types: Hydrogen bond (blue), pi-cation (orange), salt bridge (green) and hydrophobic (dark gray). DS docking score. **C**, **D** Two-dimensional ligand interaction diagrams of bictegravir and etravirine, respectively, with the residues in the binding pocket of MPXV DNA polymerase holoenzyme.

Next, we conducted a molecular docking analysis on these 50 identified compounds (Supplementary Table 4). The 20 highest-ranking drugs according to docking scores are presented in Fig. 2A, with their DEEPScreen bioactivity prediction confidence scores. Among these, seven are approved drugs (i.e., bictegravir, cefditoren, cefmenoxime, cefmetazole, elexacaftor, etravirine, and vilazodone). Interestingly, bictegravir, an integrase strand transfer inhibitor^25^ and etravirine, a non-nucleoside reverse transcriptase inhibitor^26^ are antiretroviral drugs widely used to treat HIV infection. Consequently, these two antiretroviral drugs along with two antibiotics (cefditoren and cefmetazole) were subjected to further analysis of their intermolecular interactions with MPXV DNA polymerase using the reference protein structure^27^. Bictegravir showed the highest binding affinity towards the polymerase, with a docking score (Glide SP) of –10.43 kcal/mol, followed by cefmetazole (–9.69 kcal mol^-1^), cefditoren (–7.18 kcal mol^-1^), and etravirine (–7.04 kcal mol^-1^) (Fig. 2B). Additionally, bictegravir and etravirine had analogous binding patterns (Fig. 2B). Both compounds’ chemical cores form multiple hydrogen bonds, engage pi-cation interactions with basic residues and establish van der Waals contacts with the surrounding residues. Bictegravir interacts with the MPXV DNA polymerase holoenzyme through two hydrogen bonds (SER552, and LEU553), a pi-cation interaction with LYS661, and salt bridge with ASP753 (Fig. 2C). Similarly, etravirine forms two hydrogen bonds (LYS661 and LYS826), and a pi-cation bond with ARG634c (Fig. 2D). Due to their high potential for inhibiting poxvirus DNA polymerases, these four drugs were selected for further validation in state-of-the-art human organoid models inoculated with infectious viruses.

### Structure modeling and dynamic behavior of MPXV DNA polymerase – predicted ligand complexes

We employed AF3^23^ to predict the 3D structures of protein-DNA-ligand complexes containing the predicted inhibitors, bictegravir and etravirine, to evaluate the plausibility of the binding between MPXV DNA polymerase and the indicated ligands. We obtained consistently high-confidence AF3 models for all MPXV DNA polymerase–DNA–ligand complexes (mean pLDDT: 87 and 88; and pTM: 0.89 and 0.88, for bictegravir and etravirine, respectively). Interface confidence (iPTM) values of 0.79 and 0.73 reflect expected target- and ligand-specific variation while remaining within a range supporting well-posed binding geometries. In comparison to the AF3 prediction output for the native ligand-bound (TTP) assembly from PDB id: 8HG1, these results point to stable global folds for both bictegravir and etravirine. Detailed scores are provided in Supplementary Table 2. AF3 prediction files are provided in Supplementary Data 5.

Subsequently, to observe whether the prioritized compounds maintain favorable interactions in a dynamic, solvated environment, we performed short (50 ns) molecular dynamics (MD) simulations in triplicate for the DNA polymerases of MPXV, VACV, and VARV in complex with DNA, together with the most potent compounds obtained through this study, bictegravir and etravirine, to assess the flexibility of the binding site. The VACV, and VARV DNA polymerase simulations were added as extra, to further verify their usage as surrogate viral proteins during DEEPScreen-Pox training dataset generation.

**Fig. 3:**
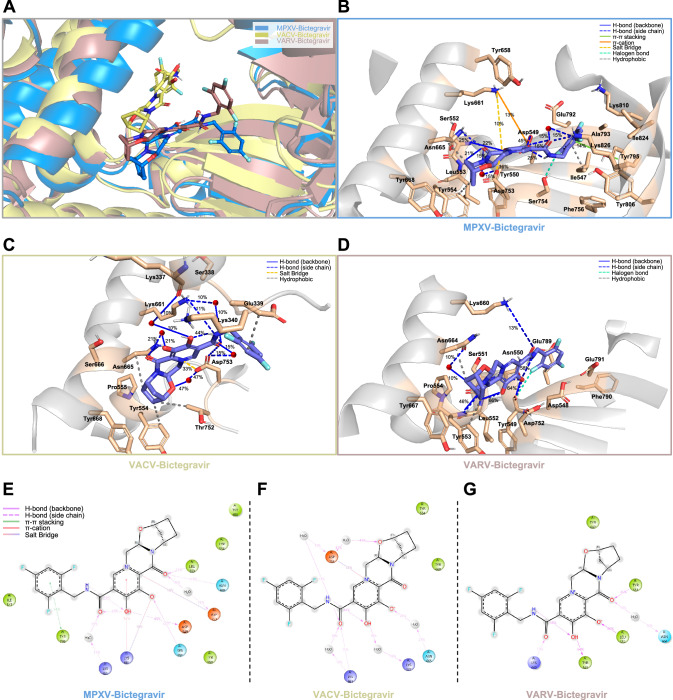
Molecular dynamics simulations plots of DNA polymerases of MPXV, VACV, and VARV against bictegravir. **A** Bictegravir was superimposed on the backbone atoms of MPXV, VACV, and VARV DNA polymerases. Polar hydrogen atoms are shown in white. The binding mode of Bictegravir within the respective targets is depicted in light blue for MPXV, mustard yellow for VACV, and brick red for VARV. The same color scheme is used to represent the DNA polymerase backbone structures, with secondary structures also highlighted in the corresponding colors. Protein sequence similarities are above 97%, and the secondary structures are folded in a highly conserved manner. Bictegravir is observed to bind in a similar fashion within the active site across all three polymerases. **B**, **C**, **D** The most populated pose of each simulation with MPXV, VACV, and VARV, respectively. Color codes explaining each interaction type are provided in the legend. **E**, **F**, **G** 2D representations of MPXV, VACV, and VARV simulations, respectively.

The overall structural stability of the enzyme was evaluated using backbone RMSD (Supplementary Fig. 3), while local fluctuations within the binding site were characterized through RMSF analysis (Supplementary Fig. 4) and the most frequently observed occupancy values were reported in Fig. 3 and Supplementary Fig. 5. The MD simulations of MPXV, VACV, and VARV DNA polymerases in complex with bictegravir reveal that residues within the palm domain (524–618) interact with the large heterocyclic core of the structure, while the fingers domain (619–675) of the enzymes interacts with the N-[(2,4,6-trifluorophenyl)methyl] group of the compound, and these interactions exhibit similar characteristics across the three polymerases. Although rotations are observed in etravirine, the interaction characteristics between the regions appear to be similar (Supplementary Fig. 5).

### Bictegravir and etravirine potently inhibit clade IIb MPXV infection in human organoids

**Fig. 4:**
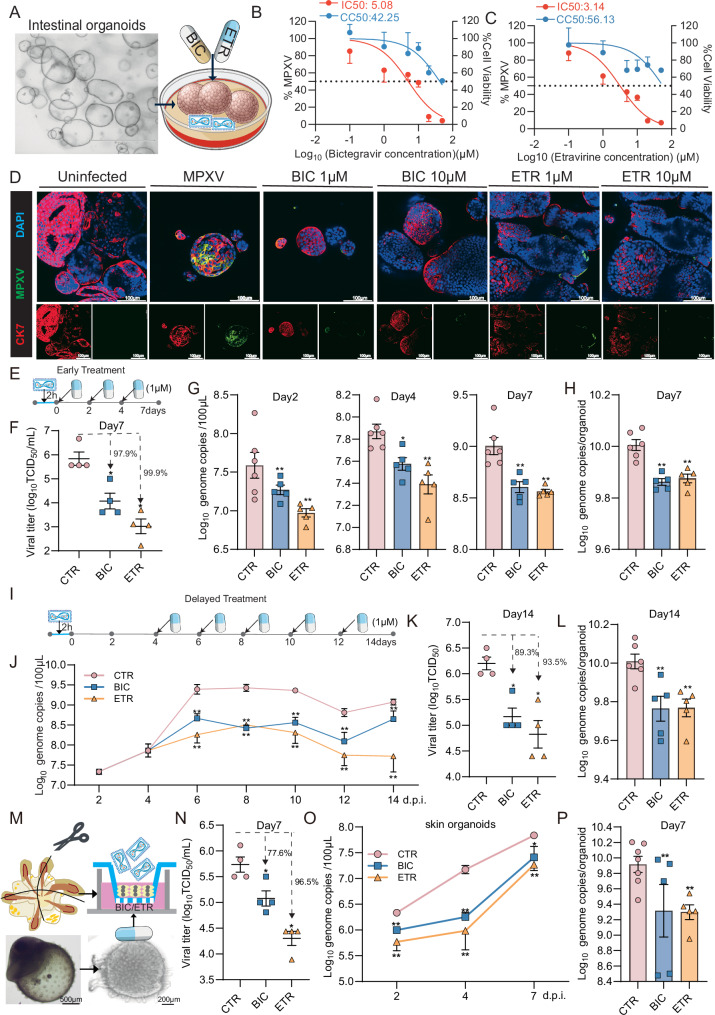
Antiviral effects of bictegravir and etravirine against clade IIb MPXV infection. **A** Schematic representation of bictegravir or etravirine treatment in human intestinal organoids. The 50% inhibitory concentration (IC50) and 50% cytotoxic concentration (CC50) of bictegravir (**B**) or etravirine (**C**) determined in clade IIb MPXV infected hIOs (*n* = 4–5). **D** Immunofluorescence staining of MPXV virions (green) and epithelial cell marker CK7 (red) in hIOs treated with different concentrations of bictegravir or etravirine (1 or 10 μM) for 48 h. Uninfected intestinal organoids incubated with the antibodies serve as the negative control. MPXV infected intestinal organoids untreated and incubated with the antibodies serve as the positive control. DAPI (blue) was applied to visualize nuclei. (Scale bar, 100 μm. 40× oil immersion objective). **E** Schematic representation of early drug treatment (1 μM) in hIOs. Quantification of MPXV infectious titers (**F**) in culture medium or viral DNA level in organoids (**H**) at 7 days with or without drug treatment (1 μM) (*n* = 4–6). **G** Quantification of MPXV DNA level in culture medium at 48 h, 96 h and 7 days with or without drug treatment (1 μM) (*n* = 5–6). **I** Schematic representation of delayed drug treatment (1 μM) in hIOs. **J** Quantification of MPXV DNA level in culture medium at 2, 4, 6, 8, 10, 12, and 14 days post-inoculation. Drug (1 µM) was administered at 4 days post-inoculation for drug treatment group (*n* = 5–6). Quantification of MPXV infectious titers in culture medium (**K**) or viral DNA level in organoids (**L**) at 14 days post-inoculation. Drug (1 µM) was administered at 4 days post-inoculation for drug treatment group (*n* = 4–6). **M** Schematic representation and illustration of MPXV infection and treatment in skin organoids cultured in ALI. Infectious viral titers in the culture medium (**N**) or viral DNA level in organoids (**P**) at 7 days post-inoculation with or without drug treatment (10 μM) (*n* = 4–7). **O** Quantification of MPXV DNA level in culture medium from organoids with or without drug treatment (10 µM) (*n* = 5–7). The schematic representations of (**A**, **E**, **I,** **M**) were created using Adobe Illustrator 2021. The bright-field images of organoids in (**A**, **M**) were captured in our experiments. Every element of these images was created by the co-authors. Data are shown as means of biological replicates ± s.e.m, ∗*p* < 0.05; ∗∗*p* < 0.01. BIC bictegravir, ETR etravirine.

During the 2022–2023 global mpox outbreak caused by the clade IIb MPXV, inflammation of the rectum (proctitis) was frequently reported^27,28^. We recently have pioneered the establishment of primary human intestinal organoids (hIOs) based models for MPXV infection^29^. To validate the antiviral activity of bictegravir, cefditoren, cefmetazole and etravirine, we inoculated hIOs with a clade IIb MPXV isolate and treated with 10 μM of the individual drugs for 48 h, with cidofovir included as a positive control (Fig. 4A and Supplementary Fig. 6). Bictegravir and etravirine significantly decreased viral DNA levels in both the supernatant and organoids compartments, and importantly the reduction of infectious viral titers was strong (Supplementary Fig. 6A–C). We extended this analysis to 96 h and 7 days treatment, confirming the potent antiviral effect of bictegravir and etravirine (Supplementary Fig. 6D–I). Considering their anti-MPXV potency but also the clinical relevance that people with HIV are particularly vulnerable to MPXV infection^13,30^, we further focused on bictegravir and etravirine.

We next profiled a series of concentrations of bictegravir and etravirine (0.1–50 μM) in hIOs infected with a 2022 clade IIb MPXV isolate from Netherlands. Based on quantification of viral DNA levels, both drugs demonstrated dose-dependent antiviral activity but high concentrations also moderately inhibited organoid growth (Fig. 4B, C, Supplementary Fig. 7A, B). Specifically, the half maximum inhibitory concentration (IC_50_) value was 5.08 and 3.14 μM, respectively (Fig. 4B, C). These effective concentrations are highly clinical relevant, since the maximum concentrations of bictegravir and etravirine have been reported to reach 4399–9611 ng/mL (equivalent to 9.79–21.39 μM) and 8261–15,652 ng mL^−1^ (18.98–35.96 μM) in the plasma of treated HIV patients, respectively^31,32^. Immunofluorescence staining further validated the potent antiviral effects of bictegravir and etravirine showing dramatically reduced levels of MPXV virions (Fig. 4D) and viral double-stranded RNA (dsRNA; formed during viral replication) (Supplementary Fig. 7C).

To recapitulate clinical treatment strategies, we first tested the scenario of early antiviral treatment in which 1 µM bictegravir or etravirine was applied 2 h after virus inoculation (Fig. 4E). Both drugs showed around 2–3 Log_10_ reduction of infectious virus production, achieving over 95% inhibition in TCID_50_ assay, a key measurement for determining bona fide antiviral activity (Fig. 4F). The viral DNA levels in culture medium at 2, 4 and 7-days and in organoids 7-days post-inoculation were significantly reduced after treatment (Figs. 3H and 4G). In addition, we also found both drugs exhibited dose-dependent antiviral activity during the early treatment (Supplementary Fig. 7D–K). Next, we also assessed the scenario of delayed treatment. We initiated the treatment with 1 µM bictegravir or etravirine at 4 days post-infection (Fig. 4I). Significant inhibition of viral DNA was observed from day 6 to 14, with about 1 Log_10_ reduction of the infectious viral titer, corresponding to 90% inhibition of infectious virus production by day 14 (Fig. 4J–L). Thus, early treatment of bictegravir and etravirine appears to be slightly more effective than delayed treatment.

Since we have developed MPXV infection models using human induced pluripotent stem cell-derived skin organoids^33^, we further validated the antiviral activity of bictegravir and etravirine in air–liquid interface (ALI)-cultured skin organoids, which can mimic human skin physiology^34^ (Fig. 4M). Bictegravir or etravirine were administered through the culture medium in the bottom of the trans-well, mimicking in vivo drug absorption. Both drugs inhibited the production of infectious virus, with etravirine achieving over 1 Log_10_ reduction of the infectious viral titer, corresponding to 96.5% inhibition (Fig. 4N). After 7 days of treatment, the viral DNA levels in culture medium (Fig. 4O) and in organoids intracellularly (Fig. 4P) were significantly reduced. Collectively, bictegravir and etravirine potently inhibit clade IIb MPXV infection in human intestinal and skin organoids.

Recently, we have developed human macrophage-augmented intestinal organoids (MaugOs), which can simultaneously recapitulate viral infection and inflammatory response^35,36^ (Supplementary Fig. 8A). Given that this model is only capable of modeling short-term infection and treatment, we performed two days treatment with bictegravir or etravirine at 10 μM. Both drugs significantly inhibited the production of infectious virus, with etravirine achieving over 1 Log10 reduction of infectious viral titer, corresponding to 95.7% inhibition (Supplementary Fig. 8B). As expected, these two antiviral drugs had no effect on the production of IL-1β cytokine, a hallmark of inflammatory response (Supplementary Fig. 8D). Consistently, immunofluorescence staining for MPXV confirmed the potent antiviral activity of both drugs in this model (Supplementary Fig. 8E).

### Bictegravir and etravirine inhibit the infections of clade Ia and Ib MPXV isolates

**Fig. 5:**
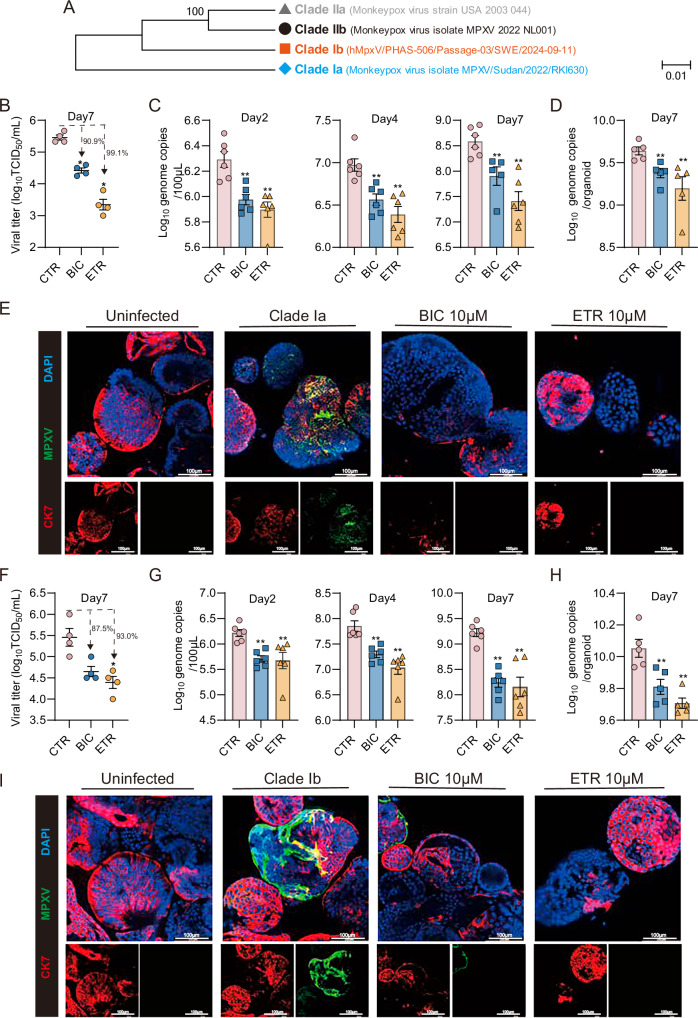
Bictegravir and etravirine inhibit the infections of clade Ia and Ib MPXV isolates in human intestinal organoids. **A** Phylogenetic tree and genetic relationships of different (sub)clades of MPXV. Quantification of clade Ia (**B**) or clade Ib (**F**) MPXV infectious titers in culture medium or clade Ia (D) or clade Ib (**H**) MPXV DNA level in organoids at 7 days with or without drug treatment (10 µM) (*n* = 4–5). Quantification of clade Ia (**C**) or clade Ib (**G**) MPXV DNA level in culture medium at 48 h, 96 h and 7 days with or without drug treatment (10 µM) (*n* = 6). Immunofluorescence staining of clade Ia (**E**) or clade Ib (**I**) MPXV virions (green) and epithelial cell marker CK7 (red) in hIOs treated with bictegravir or etravirine (10 µM) for 48 h. Uninfected intestinal organoids incubated with the antibodies serve as the negative control. Clade Ia MPXV infected intestinal organoids untreated and incubated with the antibodies serve as the positive control. DAPI (blue) was applied to visualize nuclei. (Scale bar, 100 μm. 40× oil immersion objective). Data are shown as means of biological replicates ± s.e.m, ∗*p* < 0.05; ∗∗*p* < 0.01. BIC bictegravir, ETR etravirine.

Alarmingly, the clade Ia and Ib MPXV strains are currently co-circulating in East and Central Africa and sporadic cases of clade Ib MPXV have been reported in several countries, including Sweden, Germany, the United Kingdom, India and Thailand^37–40^. Herein, we inoculated hIOs with the clade Ia and Ib isolate, respectively, to further evaluate the antiviral effects of early treatment of bictegravir and etravirine. Both drugs effectively inhibited the replication of both clade Ia and Ib MPXV strains (Fig. 5). For example, treatment with 10 μM, a clinically relevant concentration, of either drug showed around 1–2 Log_10_ reduction of infectious virus production, achieving over 85% inhibition in TCID_50_ assay (Fig. 5B, F), with etravirine achieving over 99% inhibition of clade Ia viral titer (Fig. 5B). Consistently, immunofluorescence staining for MPXV also confirmed the potent antiviral activity of both drugs (Fig. 5E, I, Supplementary Fig. 9A, B).

### Bictegravir and etravirine inhibit the infections of vaccinia virus and cowpox virus

**Fig. 6:**
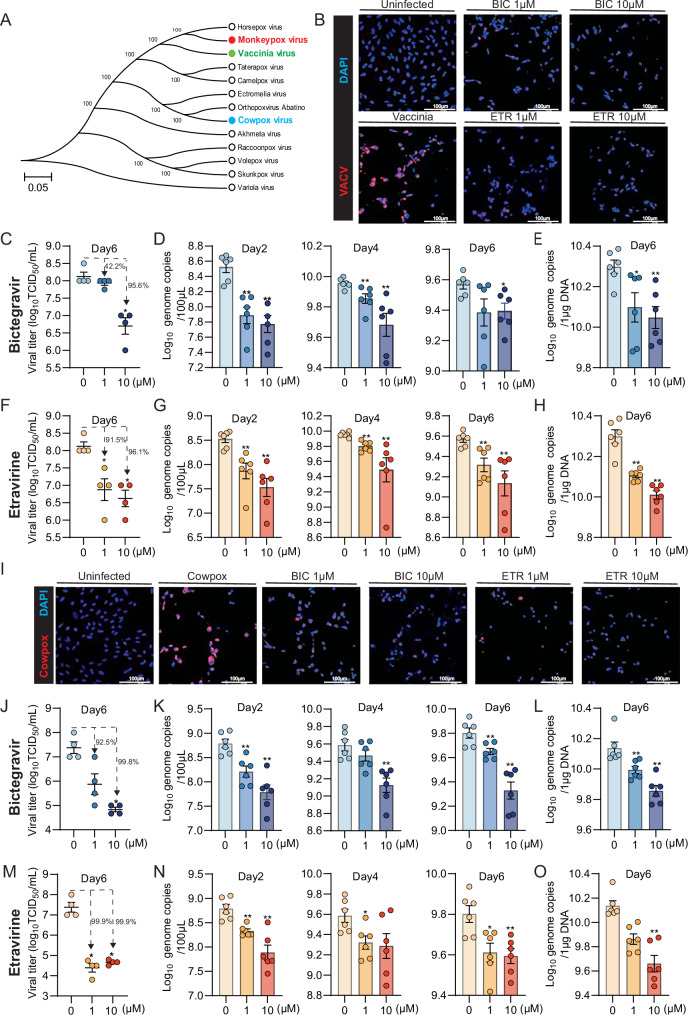
Inhibitory effects of bictegravir and etravirine on vaccinia virus and cowpox virus infections. **A** Phylogenetic tree and genetic relationships of different *Orthopoxviruses*. Immunofluorescence staining of vaccinia virus virions (red) (**B**) or cowpox virus (red) (**I**) in Vero cells treated with different concentrations of bictegravir or etravirine (1 or 10 µM) for 48 h. Uninfected cells incubated with the antibodies serve as the negative control. Vaccinia virus or cowpox virus infected cells untreated and incubated with the antibodies serve as the positive control. DAPI (blue) was applied to visualize nuclei. (Scale bar, 100 μm. 40× oil immersion objective). Quantification of infectious titers in culture medium at 6 days with or without treatment (1 or 10 μM) of bictegravir on vaccinia virus (**C**) or cowpox virus (**J**) or etravirine on vaccinia virus (**F**) or cowpox virus (**M**) (*n* = 4). Quantification of DNA level in culture medium at 48 h, 96 h and 6 days with or without treatment (1 or 10 μM) of bictegravir on vaccinia virus (**D**) or cowpox virus (**K**) or etravirine on vaccinia virus (**G**) or cowpox virus (**N**) (*n* = 6). Quantification of DNA level in Vero cells at 6 days with or without treatment (1 or 10 μM) of bictegravir on vaccinia virus (**E**) or cowpox virus (**L**) or etravirine on vaccinia virus (**H**) or cowpox virus (**O**) (*n* = 6). Data are shown as means of biological replicates ± s.e.m, ∗*p* < 0.05; ∗∗*p* < 0.01. BIC bictegravir, ETR etravirine.

The *Orthopoxvirus* genus has 13 species (Fig. 6A). Vaccinia virus is considered the prototypic poxvirus and has been widely studied^41^. To investigate whether bictegravir and etravirine possess broad-spectrum anti-poxvirus activities, we tested their early treatment effects on vaccinia virus infection in cells. Immunofluorescence staining revealed reduced numbers of infected cells following treatment with either drug (Fig. 6B). TCID_50_ assay also demonstrated a significant reduction in viral titers after treatment for 6 days, with 10 μM treatment of both drugs achieving around 1.5 Log_10_ reduction of the infectious viral titer, corresponding to over 95% inhibition (Fig. 6C, F). Similar to MPXV, both bictegravir and etravirine effectively inhibited the release of viral DNA into supernatant and suppressed viral DNA levels in infected cells (Fig. 6D, E, G, H).

Cowpox virus has an animal reservoir and is typically transmitted to humans for instance through occupational exposure to infected cows or other animals. It is a rare zoonotic infection, but can result in severe complications^42^. We inoculated cells with a cowpox virus isolate and assessed the antiviral activity of early treatment of bictegravir and etravirine. Immunofluorescence staining of virions confirmed the potent antiviral effect of both drugs against cowpox virus infection (Fig. 6I). Importantly, TCID_50_ assay demonstrated potent inhibition of infectious virus production by both drugs after 6 days treatment, with ~3log_10_ reduction by the treatment at a concentration of 10 μM (Figs. 6J, 5M). Consistently, the extracellular and intracellular viral DNA levels were significantly inhibited (Fig. 6K, L, N, O). Collectively, we demonstrated that bictegravir and etravirine possess potent and broad-spectrum antiviral activities against poxviruses, which support the robustness of our AI drug discovery pipeline.

## Discussion

Clade IIb^43^ and the newly emerged clade Ib together with clade Ia^7,8^ strains caused the 2022–2023 global outbreak and the ongoing outbreaks in Africa, respectively. During the 2022–2023 global outbreak, over 90% of the confirmed clade IIb MPXV cases were found in men who have sex with men (MSM), and a substantial proportion of these individuals were HIV co-infected^13,44–46^. Similarly, transmission of clade Ib MPXV also frequently occurs in the context of sexual contact, e.g., among sex workers and MSM^47^. HIV prevalence among female sex workers and MSM in the DRC is estimated to be 7–8%, and there are 1.3 million HIV-infected children in Africa living with HIV, with only half of them receiving antiretroviral therapy^30^. People living with HIV (PLWH) are at higher risk of MPXV infection, and advanced or untreated HIV is a strong predictor for severe mpox complications, as well as poor response to treatment^13,48^.

To better address the HIV and mpox epidemics^30,49^, we focused on the two FDA-approved antiretroviral drugs bictegravir and etravirine. Importantly, we demonstrated the potent antiviral activity of bictegravir and etravirine against all three tested MPXV strains, clade Ia, Ib and IIb. As an integrase strand transfer inhibitor, bictegravir inhibits HIV-1 replication by blocking the strand transfer step of viral DNA integration into the host genome^50^. Acting as a non-nucleoside reverse transcriptase inhibitor, etravirine blocks the RNA- and DNA-dependent DNA polymerase to inhibit HIV-1 replication^51^. In addition, owing to our AI drug discovery pipeline was designed to target the DNA polymerases of poxviruses. The docking based binding analysis showed that these two drugs bind the palm and fingers subdomains of the poxvirus DNA polymerases (Fig. 2B). Specifically, bictegravir formed hydrogen bonds with Asp549, Ser552, Leu553, and Lys661, together with a pi-cation interaction with Lys661 (Fig. 2C); etravirine formed hydrogen bonds with Asp549, Tyr550, and Lys661 and a pi-cation interaction with Arg634 (Fig. 2D). Notably, the palm and fingers subdomains position incoming nucleotides and catalyze primer extension during DNA synthesis. Thus, drug binding at these sites could impair nucleotide incorporation and genome elongation^52^. Our drug binding mode analysis provide a mechanistically coherent explanation for the observed anti-mpox efficacy observed in organoid models. Nevertheless, future research, for example using techniques such as X-ray crystallography, would be valuable in validating and further exploring the mechanistic insight.

In this study, we extensively validated the anti-poxvirus activity of bictegravir and etravirine based on the measurement of viral DNA levels, immunofluorescence staining of the virions and quantification of infectious virus titers. As the most important readout, the TCID_50_ assay consistently showed a 100–1000 fold reduction of the infectious virus titer, corresponding to over 95% inhibition after drug treatment. The drug concentrations (1 and 10 μM) that we used here are achievable in the blood of treated HIV patients^53,54^. For example, the maximum concentrations of bictegravir and etravirine have been reported to reach 4399–9611 ng/mL (equivalent to 9.79–21.39 μM) and 8261–15,652 ng/mL (18.98–35.96 μM) in the plasma of treated patients, respectively^31,32^. Consistent with its clinical manifestations^1^, we employed human skin and intestinal organoids as state-of-the-art experimental models for MPXV infection. It is arguable whether these drugs should be further validated in animal models before clinical testing. Previous studies on tecovirimat, indicate that drug efficacy data derived from animal models should be interpreted with caution. Tecovirimat was approved under the FDA’s Animal Rule for treating smallpox, based on its efficacy in animal models infected with MPXV and rabbitpox virus^55^, but did not improve clade I mpox resolution in a clinical trial in the DRC^56^. Furthermore, antiviral monotherapies are sometimes found to be suboptimal in clinical settings and are prone to induce drug resistance development. Indeed, laboratory confirmed tecovirimat-resistant MPXV strains have been detected in immunocompromised patients receiving long-term treatment with tecovirimat monotherapy^13,57^. It would be of interest to further explore the combination of bictegravir/etravirine with tecovirimat or brincidofovir (cidofovir) to achieve synergistic anti-MPXV activity and to prevent drug resistance development^12^.

Following smallpox eradication, the public is no longer routinely vaccinated against the disease. This is postulated as one of the key factors contributing to the emergence of mpox epidemics^58^. Furthermore, spillover infection of cowpox virus has been reported, especially in Europe, which can cause severe complications in infected patients^42^. Borealpox virus (formerly known as the Alaskapox virus) has recently been reported to have caused a fatal infection in an immunosuppressed patient^59^. Thus, *Orthopoxviruses* are inevitably posing an emerging threat to public health. Therefore, we took a pan-poxvirus approach to identify drug candidates with broad-spectrum antiviral activity. We have demonstrated the robustness of our strategy by successfully validating the antiviral activities of the identified drug candidates using infectious models of clade Ia, Ib and IIb MPXV, as well as vaccinia virus and cowpox virus. One limitation is that we did not have access to other poxviruses, such as borealpox, camelpox, and horsepox viruses, which should be tested in future. Another limitation is that we did not include clade IIa in our in vitro study. This is because this subclade is restricted to only a few countries in West Africa, has relatively low human-to-human transmission rates, and has not recently been a significant cause of endemic outbreaks or global spread of mpox^60^.

This project prioritized drug repurposing, thus presenting “small-scale” experimentation of screening about 11500 approved and investigational small molecule drugs. This process, including the preparation of a training dataset, training/optimizing/testing of the AI model, the prediction of binders, molecular filtering, docking analyses and expert selection, took only 7 days using a single GPU system. This process could be shortened by utilizing a more powerful infrastructure. This pipeline can be readily extended to a large number of DNA and RNA viruses, for example targeting viral replicases. The drug discovery can be scaled up, for example, through virtual screening of purchasable trillion molecules (from ChEMBL, PubChem, ZINC, MolPort, and Enamine databases), including approved/experimental drugs (from DrugBank and Pharos resources). Integrating AI and other state-of-the-art technologies such as human organoid models, would potentially revolutionize therapeutic development for pandemic response and preparedness. The moonshot 100 Days Mission for pandemic preparedness currently focuses on cutting vaccine development time for new pathogens to 100 days^61^. However, we here set the stage for achieving the ambition of identifying therapeutics within 100 days in response to epidemics. We have demonstrated that our findings and the established infrastructure bear major implications for responding to the ongoing mpox outbreak and preparing for future (poxvirus) epidemics. Our innovative approach to identifying repurposed drugs already in clinical use provides opportunities for access to effective and safe mpox treatment for especially vulnerable adults, children and pregnant women.

## Methods

### AI drug discovery pipeline development and drug candidate identification

We employed our previously developed DTI prediction system, DEEPScreen^20^, to develop the DEEPScreen-Pox model for identifying poxvirus DNA polymerase inhibitors. It is a deep learning-based framework designed to automatically predict DTI using 2D structural representations of compounds in the form of pixel-based images as input^20^. DEEPScreen replaces conventional molecular descriptors with image-based features to enhance the generalization and predictive performance of its models. By leveraging CNNS, DEEPScreen inherently learns complex molecular features from compound images (Fig. 1A), offering an accurate and efficient computational screening system for early-stage drug discovery and repurposing. DEEPScreen models comprise multiple convolutional layers, each followed by a pooling layer, and conclude with a multilayered perceptron for binary classification of molecules either as active or inactive against the target protein of interest.

To predict bioactive ligands for the MPXV DNA polymerase protein (reference protein entries in UniProtKB: Q8V523 and A0A7H0DN44), we trained the DEEPScreen-Pox model. At the time of developing this model (August, 2022), MPXV DNA polymerase has no available experimental bioactivity data in open-access resources such as ChEMBL or PubChem to be utilized as a model training dataset. To address this, we curated a training dataset using a protein family/domain-based approach, in which we collected the available experimental bioactivity data for viral DNA polymerase proteins that either contain the same ligand binding domain as MPXV DNA polymerase (i.e., DNA-directed DNA polymerase, family B”, links: https://www.ebi.ac.uk/interpro/entry/InterPro/IPR006134/) or belong to the same protein family (i.e., DNA-directed DNA polymerase, family B”; link: https://www.ebi.ac.uk/interpro/entry/InterPro/IPR006172). Ligand-binding region information was obtained from the experimentally characterized structure of the MPXV DNA polymerase holoenzyme in the Protein Data Bank (PDB) with the PDB id: 8HG1. This approach allowed us to construct the training dataset composed of 251 small molecules using relevant target protein-ligand interactions from other viral DNA polymerases, such as the prototypes of *Orthopoxvirus* (vaccinia virus and variola virus), bacteriophage T4, and the human herpesvirus (HHV) 3, 5 and 6 (Supplementary Table 1). To confirm the validity of employing other viral DNA polymerases as surrogates for MPXV, we additionally assessed their sequence, structural, and functional similarity to MPXV DNA polymerase.

With the aim of forming positive and negative training datasets, this curated drug set was split considering dose responses, with a pChEMBL value of 5.8 (approximately equivalent to 1.5 μM in terms of IC50/EC50/AC50), resulting in 79 actives and 172 inactives out of the total 251 small molecules; thus we roughly maintained a 1-to-2 ratio between positives and negatives. These molecules were split into training, validation, and test folds (80%, 10% and 10%, respectively) using the scaffold split function in the Chemprop library (https://github.com/chemprop/chemprop) to prevent model overfitting during training by ensuring that structurally similar compounds were not shared across splits. Each molecule’s Simplified Molecular Input Line Entry System (SMILES) notation was converted into an image representation with a resolution of 300×300 pixels using the RDKit library (https://github.com/rdkit/rdkit). To increase the diversity of training data and improve model generalization, data augmentation was performed. Specifically, each molecule image was rotated in 10-degree increments, resulting in 36 images per molecule(1 original and 35 augmented), which yielded 9036 data points in total. The data augmentation process conferred rotation invariance to the model, addressing a common challenge in image classification.

DEEPScreen-Pox was trained using a hyperparameter optimization approach to determine the best settings for learning rate, dropout rate, and batch size. A grid search was conducted with the following hyperparameter values; learning rate: 0.00001, 0.001, 0.01; batch size: 64, 128; and dropout: 0.2, 0.3. The optimal combination of hyperparameters was identified by considering the validation performance in MCC metric. The optimal hyperparameter values were then used to train the finalized model. The model’s overall performance was evaluated on the independent/hold-out test set.

We employed the trained DEEPScreen-Pox model (https://github.com/HUBioDataLab/DEEPScreen2) to predict potential active ligands against MPXV DNA polymerase using all drug molecule entries in the DrugBank Database (https://go.drugbank.com). Drugs that received active predictions with a ≥70% confidence score (i.e., 25 or more images -out of 36- per molecule predicted as active) were selected for further analysis.

### Sequence, structural and functional conservation analysis

Pairwise global sequence alignments between MPXV DNA polymerase against VACV and VARV DNA polymerases were performed using the EMBL-EBI EMBOSS platform (www.ebi.ac.uk/jdispatcher/psa/emboss_needle), which implements the Needleman–Wunsch algorithm^62^. The alignments were carried out with default parameters, and sequence identity percentages were calculated for both the full-length proteins and for the regions corresponding to the DNA polymerase family B domain, which includes the ligand-binding pocket. On top of sequence alignment, structural comparisons were carried out using PyMOL. The MPXV DNA polymerase–ligand complex from PDB entry 8HG1 was used to identify the binding pocket. Based on this ligand and its position in the protein, protein–ligand complexes for MPXV, VACV, and VARV were generated with AF3^23^. The resulting structures were superimposed, and binding site RMSDs were calculated for residues within 5 Å of the ligand. Finally, functional conservation among MPXV, VACV, and VARV DNA polymerases was assessed using GO annotations retrieved from the UniProtGOA database (version 226) (https://www.ebi.ac.uk/QuickGO/). For each protein pair, we identified the shared annotated GO terms. The number of shared terms was then divided by half of the total number of unique GO terms annotated to proteins 1 and 2. This yielded a functional similarity score ranging from 0 to 1, with higher values indicating greater functional similarity between DNA polymerase pairs.

### Complex structure predictions with AlphaFold3

Alphafold3 (AF3) is a foundational model for structural biology designed to predict biomolecular complex structures. As an advancement over its predecessors like Alphafold1^63^ and Alphafold2^64^, AF3^23^ improves structural accuracy for tasks such as protein-ligand co-folding by leveraging expanded training data and offering enhanced user controllability. The capability for high-fidelity structure prediction makes it a powerful tool for computational drug discovery and molecular modeling^23^. The amino acid sequences for the target proteins were obtained from the following sources: (i) MPXV DNA polymerase sequence was taken from the 8HG1^52^ crystal structure deposition in the PDB. (ii) VACV DNA polymerase sequence was obtained from PDB id 5N2E^65^. (iii) VARV DNA polymerase sequence was retrieved from UniProt accession P0DOO5^66,67^, as no experimental structure is available for the VARV protein. When predicting the complex structures, the input to the AF3 model included the corresponding binding DNA sequence (DNA Chain A: AGCTATGACCATGATTACGAATTGC, DNA Chain B: CTGCACGAATTAAGCAATTCGTAATCATGGTCATAGCT), retrieved from the PDB structure “8HG1”, along with the sequences of proteins and small molecule ligands. In separate AF3-based experiments, we employed (i) the native ligand from the reference structure “8HG1” (i.e., TTP) together with MPXV, VACV and VARV proteins, to analyze the suitability of surrogate DNA polymerases, and (ii) bictegravir and etravirine drugs, selected based on DEEPScreen-Pox DTI predictions, together with the MPXV protein, to assess the binding patterns of these potentially inhibitory ligands in the MPXV DNA polymerase. AF3 ran with default inference parameters using 10 recycling steps and sampling 5 structures per biomolecular complex (Supplementary Data 5).

### Molecular docking procedure

The crystal structure of the MPXV polymerase holoenzyme–DNA complex (PDB code “8HG1”)^52^ was utilized for molecular docking studies. This structure includes the DNA polymerase F8, the processive cofactors A22 and E4, the primer-template DNA, and an incoming dTTP substrate. Molecular docking was performed using Schrödinger Suite 2023-3, with selected compounds following the DEEPScreen-Pox bioactivity prediction analysis. Protein preparation was conducted using the Protein Preparation Wizard^68^ in Schrödinger, applying the OPLS4 force field to optimize protein structure^69^. Ligand conformations were generated using LigPrep (https://www.schrodinger.com/life-science/download/release-notes/release-2025-2/) to ensure all possible states were modeled at physiological conditions (pH 7.4 ± 1.0). A receptor grid was defined around the dTTP binding site, as specified in the PDB structure. Docking simulations were executed with Glide software, focusing on identifying optimal binding poses for each ligand. Standard Precision (GlideScore SP) mode was applied, with a van der Waals radius scaling factor of 1.0 and a partial charge cut-off of 0.25. Protein-ligand complexes were visualized using PyMOL (https://learn.schrodinger.com/public/pymol/2023-4/Content/pymol/pymol_home.htm) to examine 3D ligand-receptor interactions. Additionally, 2D interaction schemes were projected by using ACS Document 1996 styling.

### Molecular dynamics simulations

#### System preparation

The DNA polymerase enzymes of monkeypox, vaccinia, and variola viruses were selected as target proteins for MD simulations. For the monkeypox system, the crystal structure from PDB entry 8HG1 was used, which contains double-stranded DNA synthesized by DNA polymerase. The vaccinia DNA polymerase structure was obtained from PDB entry 5N2E, which was crystallized without DNA. Due to the high sequence identity between the DNA polymerases (>98%) and the sequence-independent nature of polymerase activity, double-stranded DNA from 8HG1 was incorporated into the vaccinia model following backbone-based structural alignment. For variola, no experimentally determined structure was available; therefore, a predicted three-dimensional structure was generated using AlphaFold 3^23^ with the UniProt entry P0DOO5, and DNA was incorporated using the same procedure as with vaccinia. The most potent compounds identified in this study, bictegravir and etravirine, were incorporated into the biological complexes. Bound ligand conformations were obtained using a previously applied and validated molecular docking methodology.

#### Simulation runs

All systems were subjected to short MD simulations of 50 ns in triplicate to ensure statistical reliability while maintaining feasibility for the study timeline. The simulations were carried out for each DNA polymerase (monkeypox, vaccinia, and variola) in complex with double-stranded DNA together with bictegravir and etravirine. The system was solvated with SPC water model, and neutralized with chloride ions. System parameterization employed the OPLS4 force field, and equilibration was performed using Desmond (https://www.schrodinger.com/life-science/download/release-notes/release-2025-2/). Initial preparation steps included a 2000‑step minimization with a 50 kcal/mol/Å convergence threshold under harmonic restraints on solute atoms, followed by an unrestrained minimization. The system was then equilibrated through: (i) a 12 ps NVT simulation at 10 K with heavy‑atom restraints, (ii) a 12 ps NPT simulation at 10 K with restraints, and (iii) a 24 ps NPT heating phase bringing the system temperature to 310 K. Final minimization was carried out under the NPT ensemble without restraints, using a Nose–Hoover thermostat and a Martyna–Tobias–Klein barostat. Production runs consisted of three 50 ns replicas under NPT conditions at 310 K, with trajectory snapshots and energy data recorded every 10 ps.

#### Simulation analysis

Post‑processing was conducted in Maestro, combining all replicas into a single dataset for analysis with the Simulation Interactions Diagram module. Dominant conformational states were identified via trajectory clustering in the Schrödinger Drug Discovery Suite 2025‑2, and the representative trajectory was visualized with PyMOL 3. System stability was assessed using backbone root mean square deviation (RMSD), while flexibility of individual residues, particularly within the binding site, was evaluated using root mean square fluctuation (RMSF). Additionally, ligand occupancy values were reported and visualized. Representative results are provided in Supplementary Fig. 3 (RMSD profiles), Supplementary Fig. 4 (RMSF profiles), Fig. 3 and Supplementary Fig. 5 (binding site occupancy values were retrieved from the MD simulations).

### Intestinal organoids culture

Human primary intestinal organoids were cultured using our previously established protocol. Human intestinal tissue used for research purpose in this study was approved by the Medical Ethical Council of the Erasmus MC, and informed consent was given (MEC-2021-0432; MEC-2023-0629). In brief, these organoids were cultured in organoid expansion medium (OEM), based on advanced DMEM/F12 (Invitrogen), supplemented with 1% penicillin/streptomycin (Life Technologies), 10 mM HEPES, 1xGlutamax, 1 mM N2, 1 mM B27 (all from Invitrogen), 1 μM N-acetylcysteine (Sigma) and the following growth factors: 50 ng/L mouse epidermal growth factor, 50% Wnt3a-conditioned medium and 10% noggin-conditioned medium, 20% Rspo1-conditioned medium, 10 μM nicotinamide (Sigma), 10 nM gastrin (Sigma), 500 nM A83–01 (Tocris) and 10 μM SB202190 (Sigma). The medium was refreshed every 2–3 days, and organoids were passaged 1:3 every 5–7 days.

### Skin organoid culture

We recently established MPXV infection models based on human induced pluripotent stem cell-derived skin organoids, which were used for validating the antiviral drugs in this project. Briefly, cyst-like skin organoids at days 75–90 of differentiation were cut into four and placed epidermis-up on type I-collagen-coated(2 mg mL^−1^) transwell culture insert placed on a 12-well plate containing 600 µL of Organoid Maturation Medium (OMM, composed of 49% advanced DMEM/F12, 49% Neurobasal medium (Gibco), 1× GlutaMAX (Gibco), 0.5× B2-27 minus vitamin A (Gibco), 0.5× N-2 (Gibco). After 3 weeks of ALI culture in a humidified incubator at 37 °C with 5% CO_2_, ALI-skin organoids were transferred for 6 days to an incubator without humidity at 37 °C with 5% CO_2_ (dry conditions). OMM was replaced every 2 days when cultured in humidified conditions and daily when cultured in dry conditions.

### Cell culture

Vero cells were maintained in Dulbecco’s modified Eagle medium (DMEM; Lonza) supplemented with 10% fetal calf serum (Hyclone, Logan, USA) and 100 U mL^−1^ penicillin-streptomycin. Mycoplasma negative status of these cells was confirmed through regularly testing by GATC Biotech (Konstanz, Germany). Human monocytic cell line THP-1 was cultured in RPMI 1640 Medium (Thermo Fisher) complemented with 10% (v/v) inactivated Fetal Bovine Serum with 100 IU m^−1^ L penicillin and 100 mg mL^−1^ streptomycin.

### Macrophage-augmented organoids

Establishment and characterization of macrophage-augmented organoids (MaugOs) following our previously established methods^35,36^. Briefly, when intestinal organoids reached sufficient confluence (over 75% in Matrigel) and each single organoid is ~200 μm of diameter, organoids were harvested in cold Advanced DMEM/F12 and centrifuged at 300 × *g* for 5 min to remove Matrigel. Subsequently, organoids were mechanically dissociated into small fragments. Monocyte-differentiated macrophages were then respectively mix with fragmented organoids, at a ratio of 100 organoids to 10^5^ macrophages. Diluted Matrigel from the ratio of 1:8 were pre-supplemented in culture plate for providing a supporting basement to organoids and cells. The mixture of macrophages and fragmented organoids were then seeded on the surface of diluted Matrigel.

### Virus infection and antiviral treatment

Human intestinal organoids, skin organoids cultured under ALI conditions or Vero cells were inoculated with ~5 × 10^4^ PFU of different clades of MPXV, vaccinia virus or cowpox virus and incubated at 37 °C for 2 h. After incubation, viral inoculum was removed and organoids or cells were gently washed with Phosphate-Buffered Saline (PBS) two times. For skin organoids in ALI, culture medium (600 µL per well of 24-well plate) containing serial concentrations of different drugs was then added to the bottom of the well so that it comes in contact with the dermal side of the organoid while the insert and epithelial surface of the organoid remain dry. For intestinal organoids, they were then cultured in 48-well plate supplemented with 250 μL culture medium with serial concentrations of different drugs per well. For cells, they were treated with different concentrations of drugs. For MaugOs, intestinal organoids were washed with AdDMEM/F12 medium and cultured in Matrigel for 2 days. After 2 days post-infection, organoids were harvested and integrated with macrophages for an additional 2 days treated with or without 10 μM of bictegravir or etravirine. Culture medium and organoids, cells or MaugOs were separately collected or lysed (MagNA Pure 96 External Lysis Buffer, Roche, Germany) at different time points post-inoculation for further analysis. For early treatment with antiviral drug, medium was refreshed on day 2 and day 4 post-infection, collected at day 2, day 4 and day 6 (Vero cells) or 7 (organoids), organoids or cells were lysed on day 6 or 7. In addition, delayed antiviral treatment was tested by administering 1 µM of the respective drug to end-stage organoids at 4 days post-infection. Culture medium was refreshed at 2, 4, 6, 8, 10, and 12  days, collected at 2, 4, 6, 8, 10, 12, and 14 days, organoids were lysed on 14 days post-inoculation for DNA isolation and TCID_50_ assay.

### DNA extraction and qPCR

Total DNA was purified from infected organoids, cells, supernatants or MaugOs using Macherey-Nagel NucleoSpin DNA Kit (Bioke, Netherlands) and quantified by Nanodrop ND-1000 (Wilmington, USA). Viral DNA levels were quantified by SYBR Green-based qRT-PCR (Applied Biosystems SYBR Green PCR Master Mix; Thermo Fisher Scientific Life Sciences) with the StepOnePlus System (Thermo Fisher Scientific Life Sciences). Primers used in this study are listed in Supplementary Table 5.

### Quantification of virus genome copy numbers

Purified viruses were used to isolate DNA and served as a template for quantifying genome copy number. Primers used to quantify MPXV are: (Forward-GGCTCTTCTATCAACCACA; Reverse-AGTCATTATCTCCTCCTCCA). Ten-fold serial dilutions of viral DNA from 10^-1^ to 10^-8^ were prepared and quantified by qRT-PCR to generate a standard curve, as formula “y  =   − 0.278x  +  13.744” (*R*^2^ = 0.9967). Primers used to quantify vaccinia virus and cowpox virus are: (Forward- CATCATCTGGAATTGTCACTACTAAA; Reverse ACGGCCGACAATATAATTAATGC). Ten-fold serial dilutions of vaccinia virus DNA from 10^−3^ to 10^−8^ were prepared and quantified by qRT-PCR to generate a standard curve, as formula “y  =   − 0.2966x  +  13.747” (*R*^2^ = 0.9913). Ten-fold serial dilutions of cowpox virus DNA from 10^−4^ to 10^−9^ were prepared and quantified by qRT-PCR to generate a standard curve, as formula “y  =   − 0.3207x  +  14.344” (*R*^2^ = 0.9921). Virus copy numbers were calculated as follows: Copy number (molecules μL^−1^) = [concentration(ng μL^-1^) × 6.022 × 10^23^ (molecules mol^−1^)]/[DNA length × 660 (g mol^−1^) × 10^9^(ng g^−1^)].

### Immunofluorescence staining

hIOs or Vero cells cultured in the 8 μ-slide well chamber (cat. no. 80826; ibidi GmbH) were inoculated with ~5 × 10^4^ PFU viruses at 37 °C for 2 h. The culture medium was then replaced by medium containing different concentrations of antiviral drugs and they were cultured for another 48 h. For MaugOs, hIOs were inoculated with ~5 × 10^4^ PFU of clade 2b MPXV at 37 °C for 2 h. Organoids were washed with AdDMEM/F12 medium and cultured in Matrigel for 2 days. After 2 days post-infection, organoids were harvested and integrated with macrophages for an additional 2 days treated with or without 10 μM of bictegravir or etravirine in the 8 μ-slide well chamber (cat. no. 80826; ibidi GmbH). Organoids or MaugOs were washed in cold advanced DMEM/F12 medium 3 times to remove all basal matrix. Then hIOs, cells or MaugOs grown on 8 μ-slide well were fixed with 4% (w/v) paraformaldehyde (PFA) overnight at 4 °C. Organoids or cells were then rinsed 3 times with PBS for 5 min each time, followed by permeabilizing with PBS containing 0.2% (vol/vol) tritonX100 for 15 min. hIOs were added into the CytoSpin II Cytocentrifuge (Shandon Scientifi Ltd, Runcorn, England) and spun down into slides at 1000 rpm for 5 min. Then the slides or plates were twice rinsed with PBS for 5 min, followed by incubation with blocking solution (5% donkey serum, 1% bovine serum albumin, 0.2% tritonX100 in PBS) at room temperature for 1 h. Next, slides or plates were incubated in a humidity chamber with primary antibody diluted in blocking solution at 4 °C overnight. Primary antibodies used in this study are as follows: rabbit polyclonal anti-Vaccinia virus Lister Strain (FITC) (1:250, rabbit mAb), anti-Vaccinia virus Lister Strain (1:250, rabbit mAb), anti-dsRNA antibody (1:200, mouse mAb), anti-CK7 (1:300, mouse, mAb), anti-EpCAM antibody (1:500, rabbit mAb). Excess primary antibodies were removed, and the slides or plates were washed 2 times for 10 min each in PBS containing 0.2% (vol/vol) tritonX100 and once for 10 min each in PBS prior to 1 h incubation with 1:1000 dilutions of the anti-mouse IgG (H + L, Alexa Fluor® 594), the anti-rabbit IgG (H + L, Alexa Fluor® 488, which was only used for anti-EpCAM antibody) and goat anti-rabbit IgG (H + L, Alexa Fluor® 647). Nuclei were stained with DAPI (4, 6-diamidino-2-phenylindole; Invitrogen). Images were detected using Leica SP5 cell imaging system.

### TCID_50_ assay

Viruses in the cultured cells and the supernatant were harvested through repeated freezing and thawing for three times. Virus titers were quantified by using a 50% tissue culture infectious dose (TCID_50_) assay. Briefly, ten-fold dilutions of MPXV, vaccinia virus or cowpox virus were inoculated onto Vero cells, grown in 96-well tissue culture plates at 2000 cells per well. The plate was incubated at 37 °C for 5–7 days, and each well was examined under a light microscope for cytopathic effect. The TCID_50_ value was calculated by using the Reed-Muench method.

### AlamarBlue assay

Culture supernatant was discarded, and the organoids were incubated with a 1:20 dilution of AlamarBlue regent (Invitrogen, DAL1100) in culture medium for 2 h at 37 °C. Subsequently, 100 μL medium was collected to assess cell metabolic activity, with each sample being measured in duplicate. Absorbance measurements were obtained using a fluorescence plate reader (CytoFluor Series 4000, PerSeptive Borganoidsystems) at an excitation wavelength of 530/25 nm and an emission wavelength of 590/35 nm.

### Statistics and reproducibility

All statistical analyses were completed using GraphPad Prism 8.0. Comparison between two groups was analyzed by Mann–Whitney U test. Data are presented as mean ± standard error of the mean (s.e.m.). *P*  <  0.05 was considered statistically significant. For immunofluorescence staining, at least two biological replicates were prepared, and representative images were selected.

### Reporting summary

Further information on research design is available in the Nature Portfolio Reporting Summary linked to this article.

## Supplementary information

Supplementary Information

Description of Additional Supplementary Materials

Supplementary Data 1

Supplementary Data 2

Supplementary Data 3

Supplementary Data 4

Supplementary Data 5

Reporting Summary

## Data Availability

The datasets used and/or analyzed and model results of the current study are available on Mendeley Data^70^ (https://data.mendeley.com/datasets/hnfpf64n6g/1) (e.g., source values underlying Fig. 4 can be found in Files, Data, Fig. 4).

## Code availability

The framework DEEPScreen-Pox is publicly available on GitHub https://github.com/HUBioDataLab/DEEPScreen2 in the form of a Jupyter Notebook (under GPL3.0 license) (https://github.com/HUBioDataLab/DEEPScreen2). All necessary files are included in the repository. The files under DEEPScreen2/druggen/contain the results used in this study.
