# Supplementary material for: AI-driven discovery of antiretroviral drug bictegravir and etravirine as inhibitors against monkeypox and related poxviruses
Source: Commun Biol. 2025 Dec 2;8:1734. doi: 10.1038/s42003-025-09129-x (PMC12673143; doi:10.1038/s42003-025-09129-x)
Supplement: Supplementary file 1 — Supplementary Information [file 42003_2025_9129_MOESM1_ESM.pdf]

Supplementary Information for

AI-driven discovery of antiretroviral drug bictegravir and etravirine as inhibitors against monkeypox and related poxviruses

Yining Wang[1,2#], Atabey Ünlü[3,4#], Xin Wang[2#], Elif Çevrim[3,4#], Dewy M. Offermans[2], Myrthe P. Flesseman[5,6], Luca M. Zaeck[7], Liping Wu[2], Marcel J. C. Bijvelds[2], Nadia A. Sam-Agudu[8,9,10], Rory D. de Vries[7], Karine Raymond[5,6,11], Pengfei Li[2], Abdurrahman Olğaç[12], Wenshi Wang[13*], Tunca Doğan[3,4,14*], Qiuwei Pan[2*]

1. School of Life Sciences and Biopharmaceutical Sciences, Shenyang Pharmaceutical University, Shenyang 110016, China.
2. Department of Gastroenterology and Hepatology, Erasmus MC-University Medical Center, Rotterdam, the Netherlands.
3. Biological Data Science Lab, Department of Computer Engineering, Hacettepe University, 06800, Ankara, Turkey.
4. Department of Bioinformatics, Graduate School of Health Sciences, Hacettepe University, 06800, Ankara, Turkey.
5. Department of Anatomy and Embryology, Leiden University Medical Center, Leiden, Netherlands.
6. The Novo Nordisk Foundation Center for Stem Cell Medicine (reNEW), Leiden University Medical Center, Leiden, Netherlands.
7. Department of Viroscience, Erasmus MC-University Medical Center, Rotterdam, the Netherlands.
8. International Research Center of Excellence, Institute of Human Virology Nigeria, Abuja, Nigeria.

9. Department of Pediatrics and Child Health, School of Medical Sciences, University of Cape Coast, Cape Coast, Ghana
10. Global Pediatrics Program and Division of Infectious Diseases, Department of Pediatrics, University of Minnesota Medical School, Minneapolis, MN, United States.
11. University of Grenoble Alpes, CEA, Inserm, IRIG, UA13 BGE, Biomics, Grenoble, France.
12. Department of Pharmaceutical Chemistry, Faculty of Pharmacy, Gazi University, 06560, Ankara, Turkey.
13. Department of Pathogen Biology and Immunology, Jiangsu Key Laboratory of Immunity and Metabolism, Jiangsu International Laboratory of Immunity and Metabolism, Xuzhou Medical University, Xuzhou 221004, China.
14. Department of Health Informatics, Institute of Informatics, Hacettepe University, 06800, Ankara, Turkey.

[#]These authors contributed equally.

*These authors jointly supervised this work: q.pan@erasmusmc.nl (Q.P.); tuncadogan@gmail.com (T.D.); wenshi.wang@xzhmu.edu.cn (W.W.)

This file contains:

Supplementary Figure 1 to 9;

Supplementary Table 1 to 5;

References.

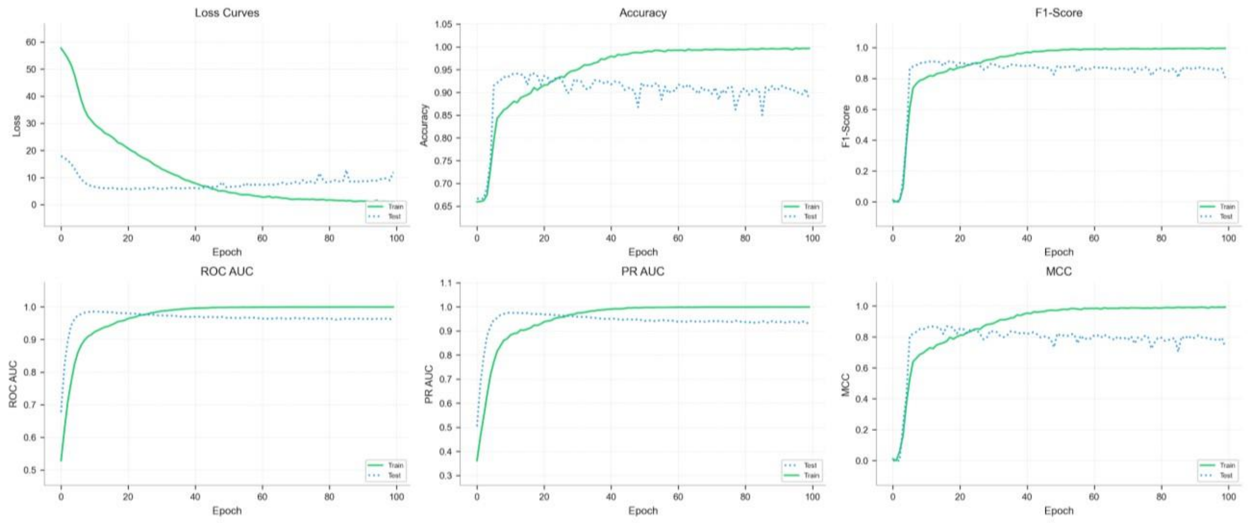

Supplementary Figure 1. Loss and model performance curves on training and testing datasets in the training of the optimal DEEPScreen MPXV DNA polymerase drug-target interaction prediction model.

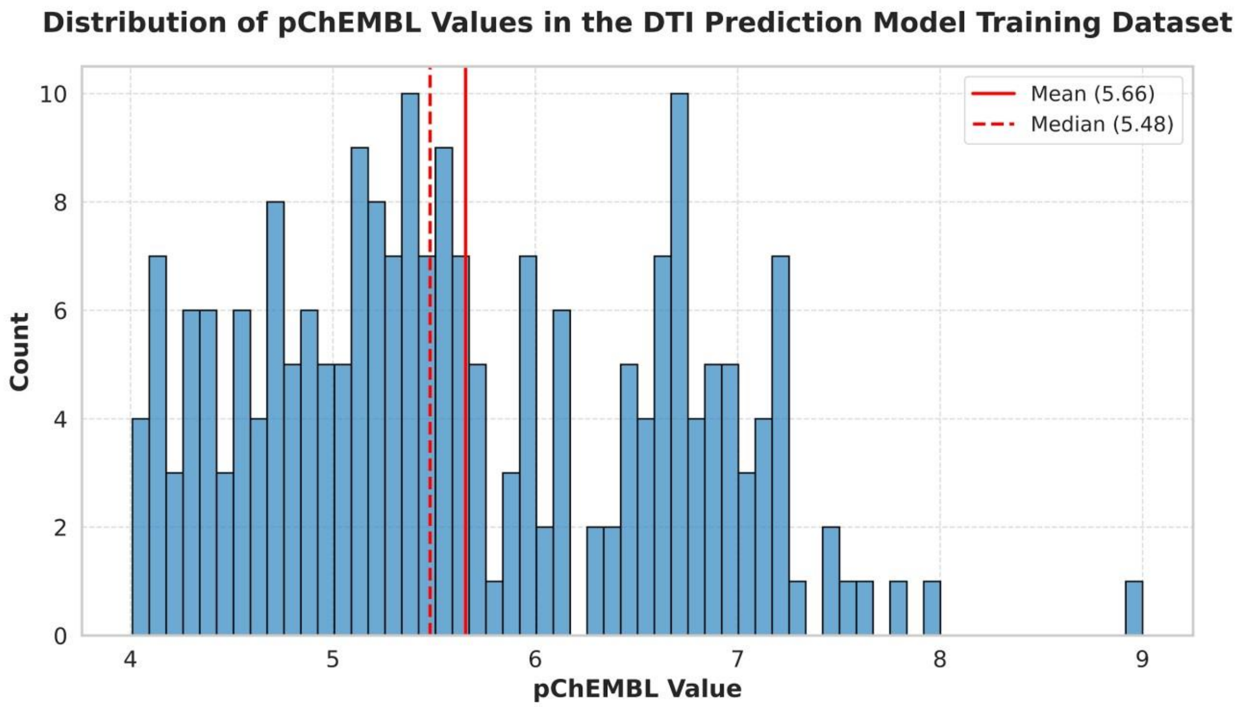

Supplementary Figure 2. Bioactivity value distribution of the 251 ChEMBL small molecules that comprise the training dataset of the DEEPScreen MPXV DNA polymerase drug-target interaction prediction model.

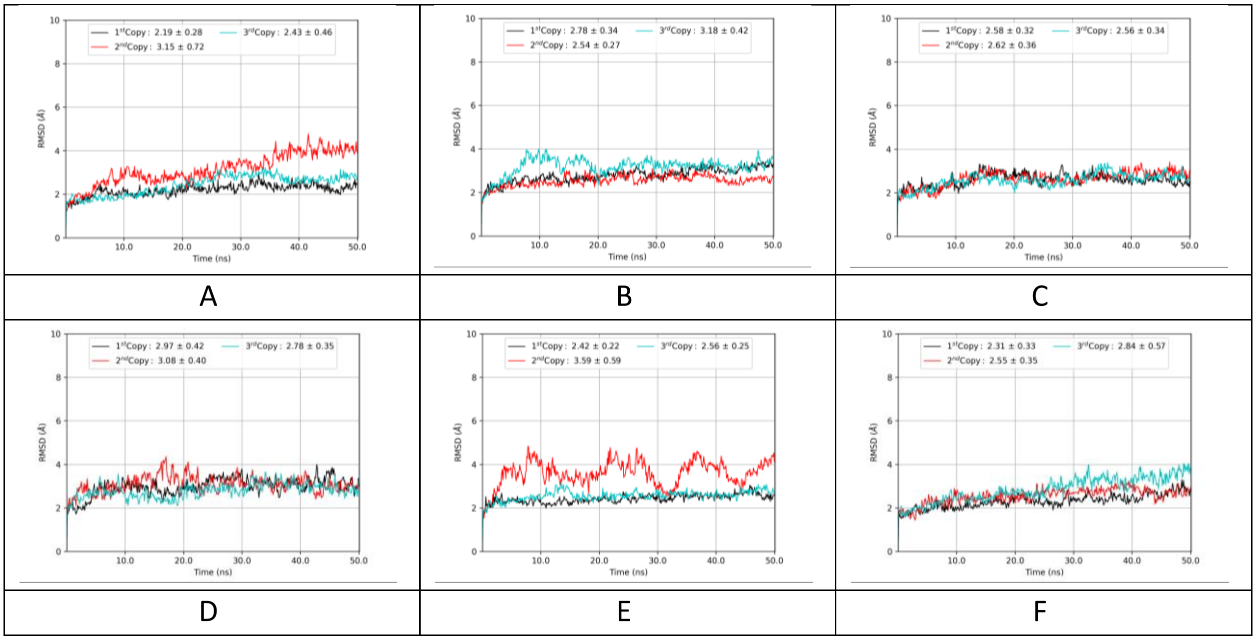

Supplementary Figure 3. RMSD plots of the DNA polymerase backbones of monkeypox, vaccinia, variola viruses simulated with bictegravir (A-C, respectively) and etravirine (D-F, respectively).

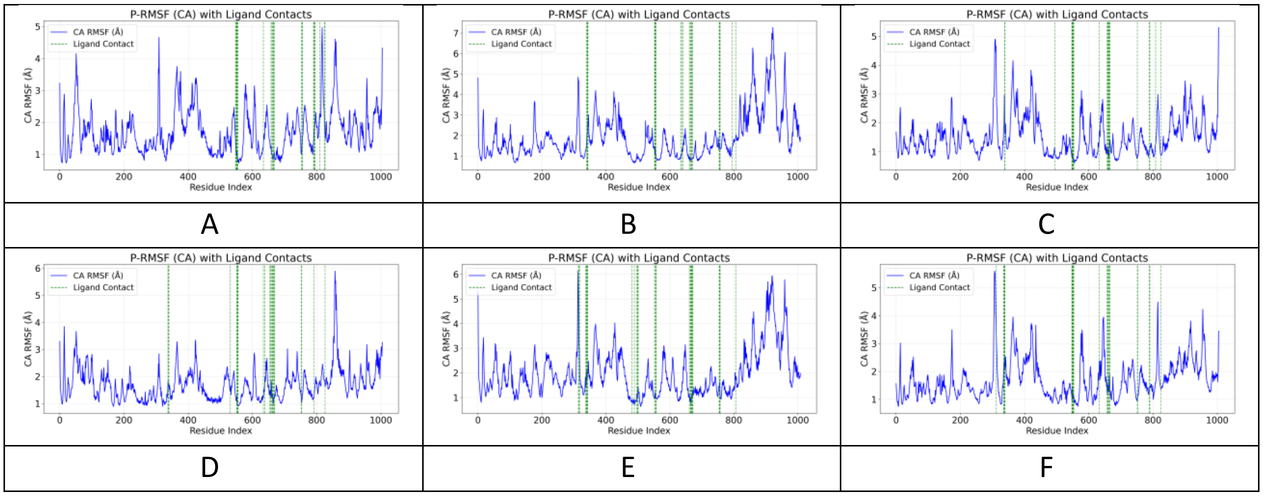

Supplementary Figure 4. Plots of the Root Mean Square Fluctuation (RMSF) of Cα atoms from DNA polymerase simulations of the monkeypox, vaccinia, and variola viruses, in complex with bictegravir (A–C, respectively) and etravirine (D–F, respectively).

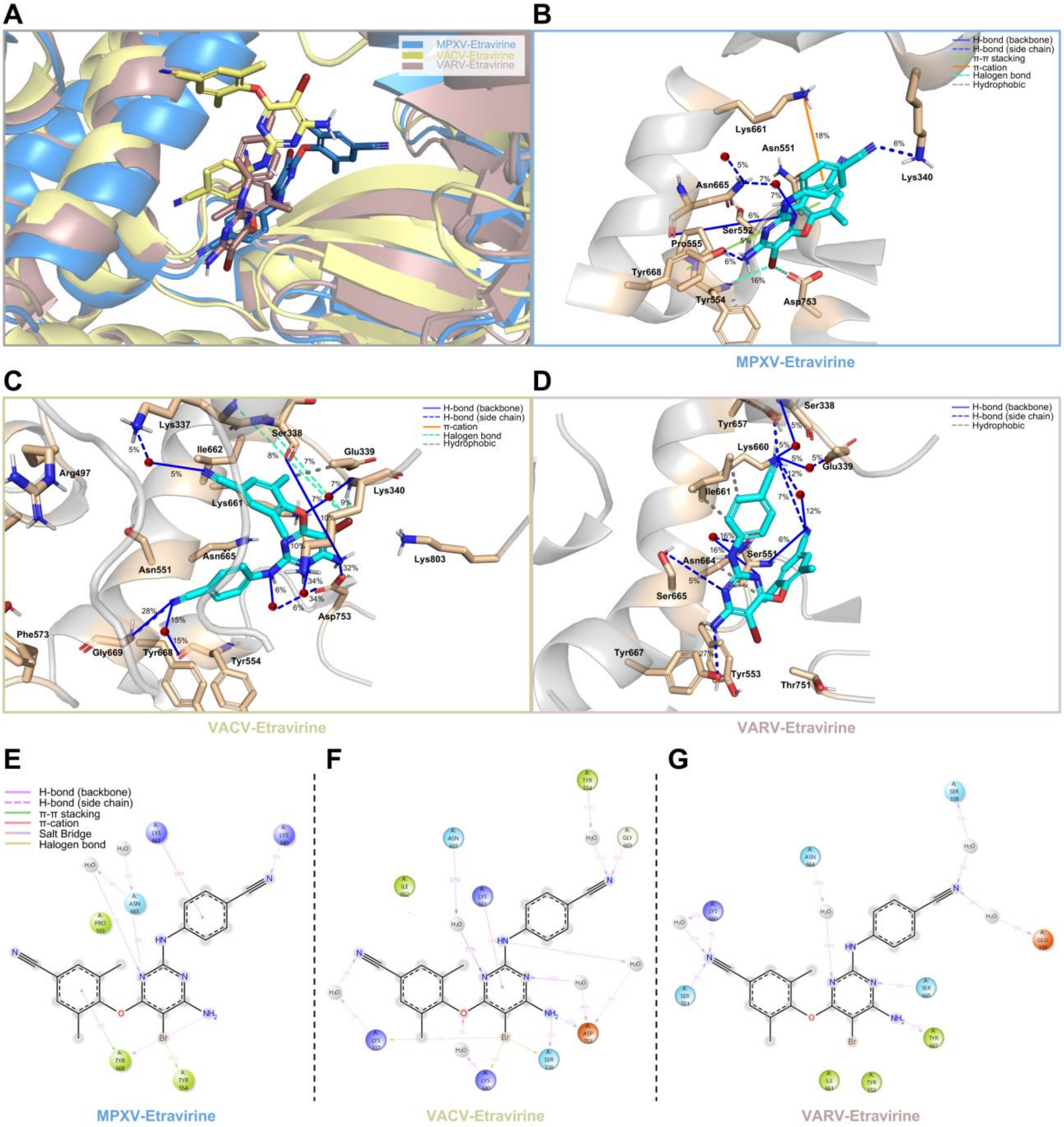

Supplementary Figure 5. Molecular dynamics simulations plots of DNA polymerases of MPXV, VACV, and VARV against etravirine. (A) Etravirine was superimposed on the backbone atoms of MPXV, VACV, and VARV DNA polymerases. Polar hydrogen atoms are shown in white. The binding mode of etravirine within the respective targets is depicted in light blue for MPXV, mustard yellow for VACV, and brick red for VARV. The same color scheme is used to represent the DNA polymerase backbone structures, with secondary structures also highlighted in the corresponding colors. Etravirine is observed to bind in a similar fashion within the binding site across all three polymerases. **(B)**, **(C)**, and **(D)** The most populated pose of each simulation with MPXV, VACV, and

VARV, respectively. Color codes explaining each interaction type are provided in the legend. **(E)**, **(F)** and **(G)** 2D representations of MPXV, VACV, and VARV simulations, respectively.

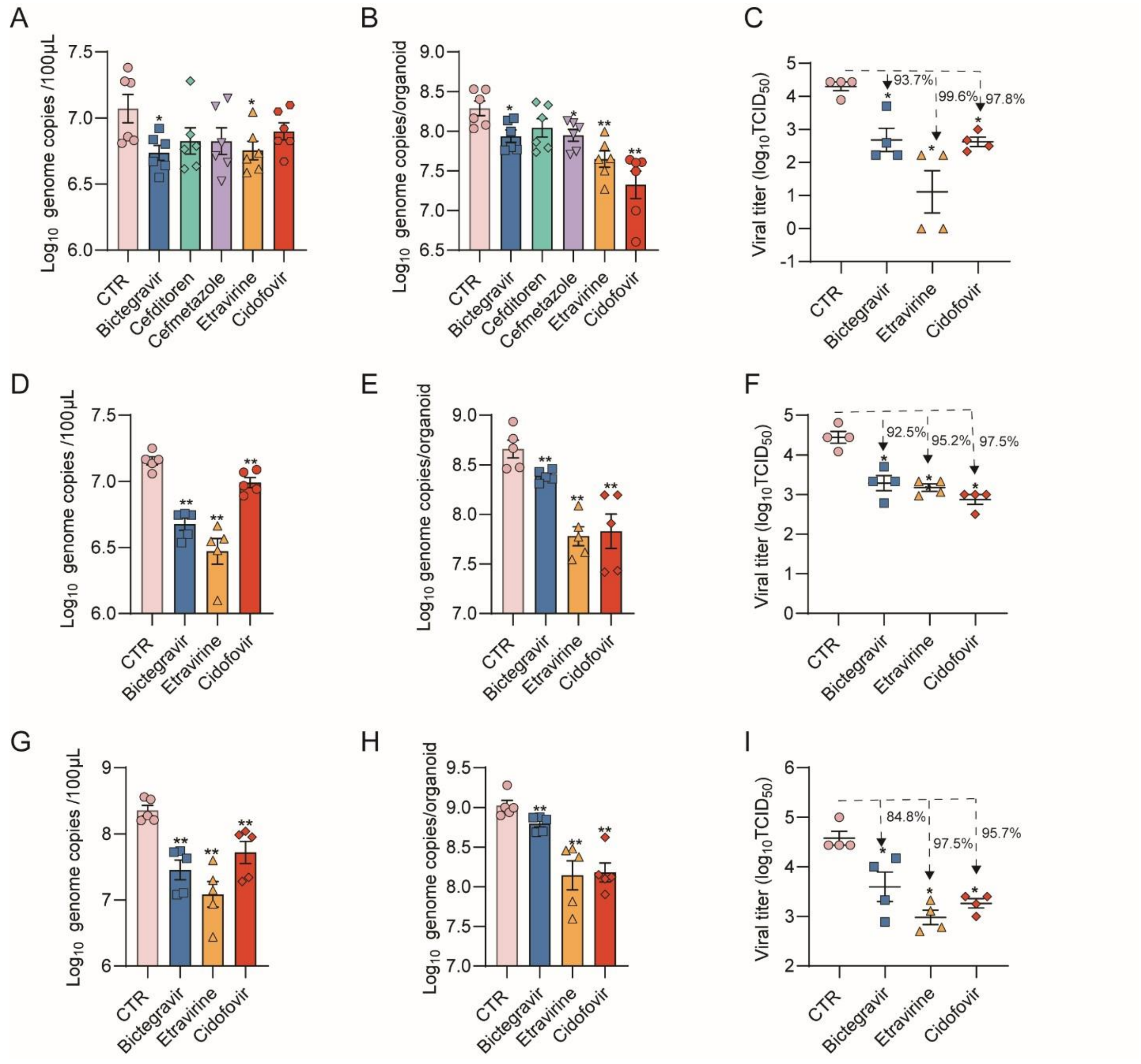

Supplementary Figure 6. Drug profiling showing the antiviral activities of bictegravir and etravirine in human intestinal organoids infected with a clade IIb MPXV isolate. A, D, G, Quantification of MPXV DNA level in culture medium at 48 hours **(A)**, 96 hours **(D)** and 7 days **(G)** after 10 µM drug treatment (n = 5-6). **B, E, H,** Quantification of MPXV DNA level in organoids at 48 hours **(B)**, 96 hours **(E)** and 7 days **(H)** after 10 µM drug treatment (n = 5-6). **C, F, I,** Quantification of MPXV infectious titres in culture medium at 48 hours **(C)**, 96 hours **(F)** and 7 days **(I)** after 10 µM drug treatment (n = 4). Data are shown as means of biological replicates ± s.e.m, ∗ $p < 0.05$; ∗∗$p < 0.01$. BIC, bictegravir; ETR, etravirine.

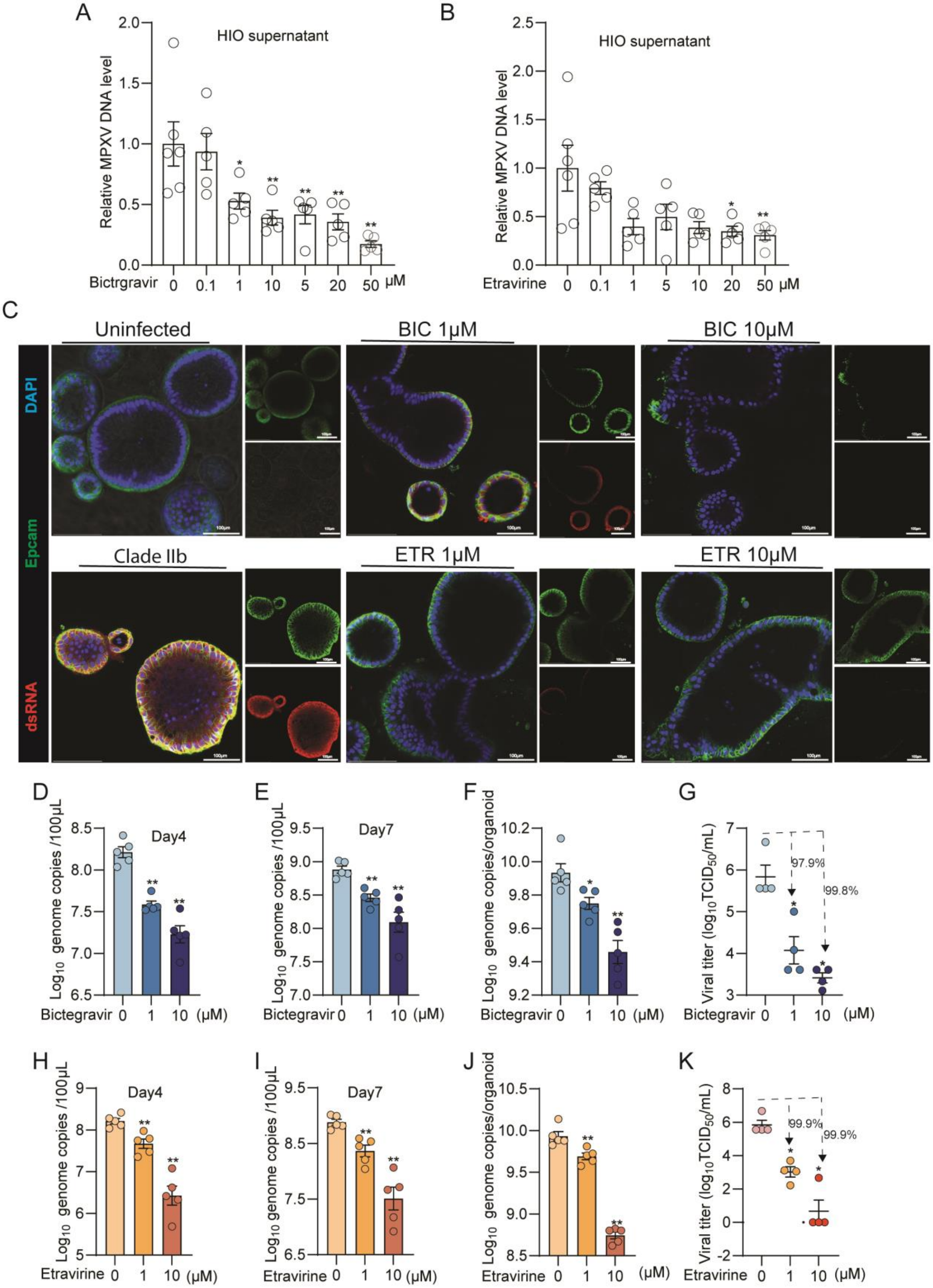
A
HIO supernatant
Relative MPXV DNA level
Bictrgravir 0 0.1 1 10 5 20 50 μM
B
HIO supernatant
Relative MPXV DNA level
Etravirine 0 0.1 1 5 10 20 50 μM
C
Uninfected
BIC 1μM
BIC 10μM
Clade IIb
ETR 1μM
ETR 10μM
DAPI
Epcam
dsRNA
D
Day4
Log10 genome copies /100μL
Bictegravir 0 1 10 (μM)
E
Day7
Log10 genome copies /100μL
Bictegravir 0 1 10 (μM)
F
Log10 genome copies/organoid
Bictegravir 0 1 10 (μM)
G
Viral titer (log10TCID50/mL)
97.9%
99.8%
Bictegravir 0 1 10 (μM)
H
Day4
Log10 genome copies /100μL
Etravirine 0 1 10 (μM)
I
Day7
Log10 genome copies /100μL
Etravirine 0 1 10 (μM)
J
Log10 genome copies/organoid
Etravirine 0 1 10 (μM)
K
Viral titer (log10TCID50/mL)
99.9%
99.9%
Etravirine 0 1 10 (μM)

Supplementary Figure 7. Bictegravir and etravirine exerted dose-dependent antiviral activity against clade IIb MPXV infection. A, B, Dose-dependent inhibition of MPXV replication in culture medium of hIOs by bictegravir **(A)** or etravirine **(B)** treatment (n = 5). **C**, Immunofluorescence staining of virus dsRNA (red), epithelial cell marker Epcam (Green) and DAPI nuclei staining (blue) in hIOs. (Scale bar, 100 µm. 40× oil immersion objective). **D, E,** Quantification of MPXV DNA level in culture medium at 96 hours **(D)** and 7 days **(E)** after 1 or 10 µM bictegravir treatment (n = 5). **F**, Quantification of MPXV DNA level in organoids at 7 days after 1 or10 µM bictegravir treatment (n = 5). **G**, Quantification of MPXV infectious titres in culture medium at 7 days after 1 or 10 µM bictegravir treatment (n = 4). **H, I,** Quantification of MPXV DNA level in culture medium at 96 hours **(H)** and 7 days **(I)** after 1 or 10 µM etravirine treatment (n = 5). **J**, Quantification of MPXV DNA level in organoids at 7 days after 1 or10 µM bictegravir treatment (n = 5). **K**, Quantification of MPXV infectious titres in culture medium at 7 days after 1 or 10 µM bictegravir treatment (n = 4). Data are shown as means of biological replicates ± s.e.m, $*p < 0.05$; $**p < 0.01$. BIC, bictegravir; ETR, etravirine.

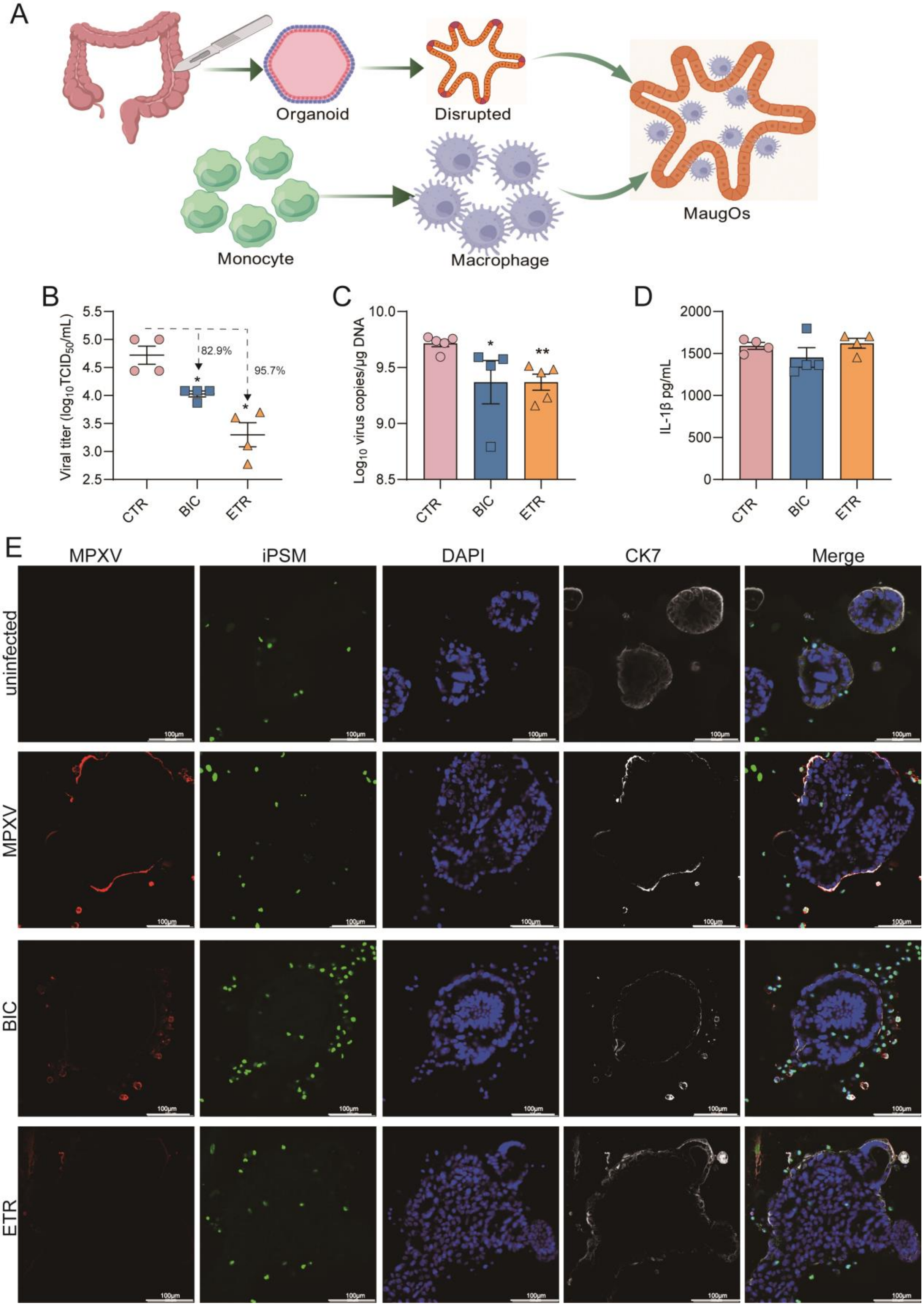
A
Organoid
Disrupted
MaugOs
Monocyte
Macrophage
B
Viral titer ($log_{10}TCID_{50}$/mL)
82.9%
95.7%
CTR
BIC
ETR
C
Log_{10} virus copies/µg DNA
D
IL-1β pg/mL
E
MPXV
iPSM
DAPI
CK7
Merge
uninfected
MPXV
BIC
ETR
100µm

Supplementary Figure 8. The effects of bictegravir and etravirine against clade IIb MPXV infection in MaugOs model. (A) Schematic overview of constructing macrophage-augmented intestinal organoids (MaugOs), created using BioGDP.com[1]. (**B)** Quantification of infectious titers in culture medium of MaugOs at 2 days with or without 10 µM of bictegravir or etravirine (n = 4). (**C)** Quantification of DNA level in MaugOs at 2 days with or without treatment 10 µM of bictegravir or etravirine (n = 4-5). (**D)** Quantification of IL-1β cytokine production by ELISA in culture medium of MaugOs at 2 days with or without treatment 10 µM of bictegravir or etravirine (n = 4). **(E)** Immunofluorescence staining of MPXV virions (red), Macrophages (green) and epithelial cell marker CK7 (white) in hIOs treated with 10 µM of bictegravir or etravirine for 2 days. Uninfected MaugOs incubated with the antibodies serve as the negative control. MPXV infected MaugOs untreated and incubated with the antibodies serve as the positive control. DAPI (blue) was applied to visualize nuclei. (Scale bar, 100 µm. 40× oil immersion objective). Data are shown as means of biological replicates ± s.e.m, $*p < 0.05$; $**p < 0.01$. BIC, bictegravir; ETR, etravirine.

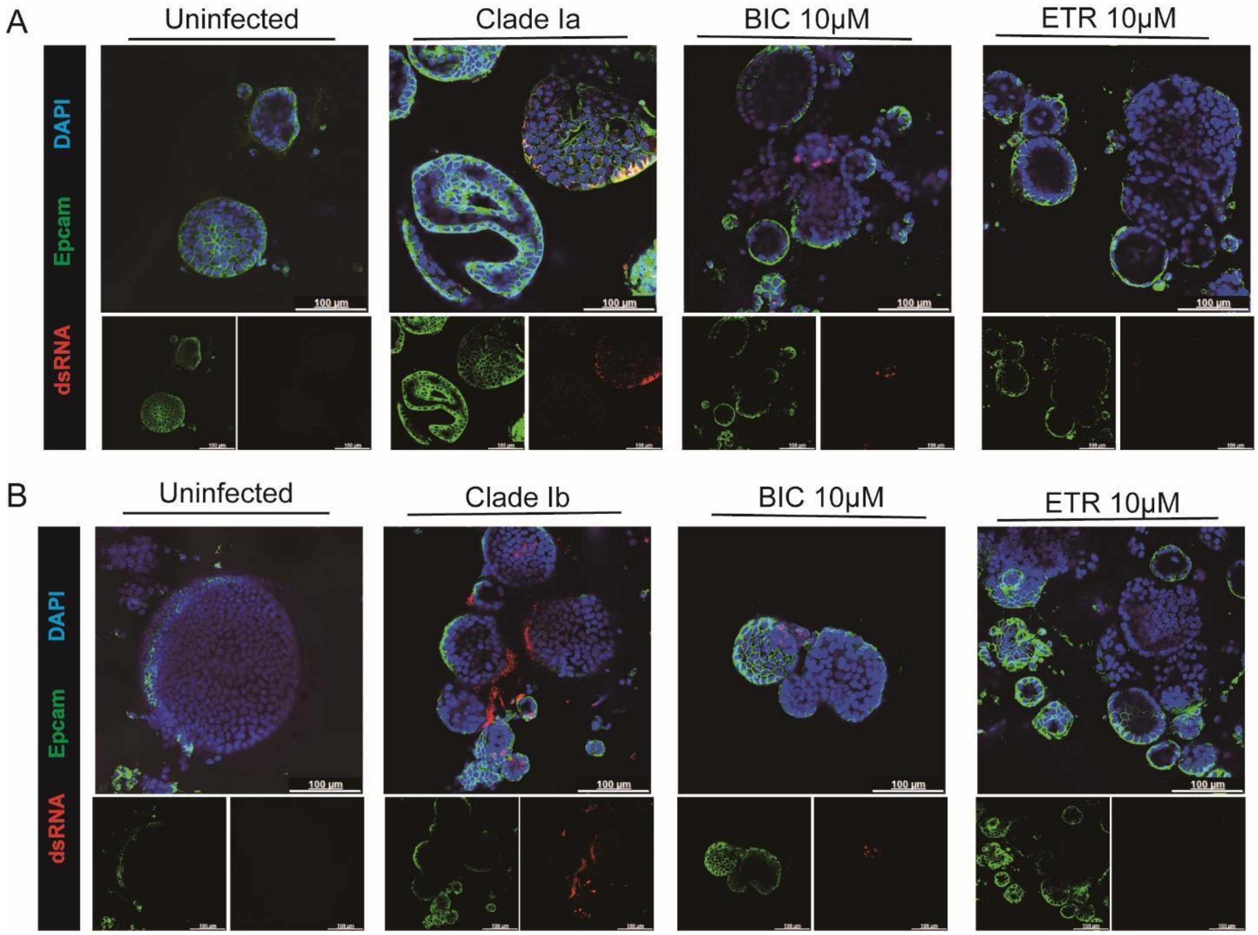

Supplementary Figure 9. Immunofluorescence staining confirmed the antiviral activity of bictegravir and etravirine in clade Ia and Ib MPXV isolates. (A and B) Immunofluorescence staining of virus dsRNA (red), epithelial cell marker Epcam (Green) and DAPI nuclei staining (blue) in clade Ia **(A)** or Ib **(B)** MPXV infected hIOs. (Scale bar, 100 μm. 40× oil immersion objective). BIC, bictegravir; ETR, etravirine.

Supplementary Table 1. Relevant viral DNA polymerase proteins used in the DEEPScreen-Pox training dataset.

Virus	Taxonomic information
Vaccinia virus (strain Western Reserve) (VACV) (Vaccinia virus – strain WR)	https://www.uniprot.org/taxonomy/10254
Variola virus	https://www.uniprot.org/taxonomy/10255
Enterobacteria phage T4	https://www.uniprot.org/taxonomy/10665
Varicella-zoster virus (strain Dumas) (HHV-3) (Human herpesvirus 3)	https://www.uniprot.org/taxonomy/10338
Human cytomegalovirus (strain AD169) (HHV-5) (Human herpesvirus 5)	https://www.uniprot.org/taxonomy/10360
Human herpesvirus 1 (strain KOS) (HHV-1) (Human herpes simplex virus1)	https://www.uniprot.org/taxonomy/10306
Human alphaherpesvirus 2	https://www.uniprot.org/taxonomy/10310
Human betaherpesvirus 5	https://www.uniprot.org/taxonomy/10359
Hepacivirus hominis	https://www.uniprot.org/taxonomy/3052230
Herpes simplex virus (type 1 / strain 17)	https://www.uniprot.org/taxonomy/10299
Human herpesvirus 6A (strain Uganda-1102) (HHV-6 variant A) (Human Blymphotropic virus)	https://www.uniprot.org/taxonomy/10370

Supplementary Table 2. AlphaFold3 (AF3) confidence metrics iPTM, pTM, and mean pLDDT, for complexes: monkeypox (MPXV), vaccinia (VACV), and variola (VARV) virus DNA polymerase proteins with the TTP ligand (native ligand in PDB id: 8HG1) (top block), and MPXV against TTP, bictegravir and etravirine (bottom block), together with DNA chains.

**Complex (Protein-DNA-Ligand)**	**iPTM**	**PTM**	**Mean pLDDT**
MPXV-DNA-TTP	0.89	0.91	88.14
VACV-DNA-TTP	0.90	0.92	88.26
VARV-DNA-TTP	0.91	0.92	88.73
MPXV-DNA-TTP	0.89	0.91	88.14
MPXV-DNA-Bic	0.79	0.89	86.82
MPXV-DNA-Etr	0.73	0.88	87.87

Supplementary Table 3. Gene Ontology (GO) annotations of MPXV, VACV, and VARV DNA polymerases obtained from the UniProtGOA Database (version 226), accessed via QuickGO browser, highlighting shared functional terms.

Protein IDs	Protein Name	GO Term id	GO Term name	GO Term category	Evidence code (ECO ID)
A0A7H0DN44 (MPXV), P20509 (VACV), P0DOO5 (VARV)	DNA polymerase	GO:0000166	nucleotide binding	F*	ECO:0000256
A0A7H0DN44 (MPXV), P20509 (VACV), P0DOO5 (VARV)	DNA polymerase	GO:0003676	nucleic acid binding	F	ECO:0000256
A0A7H0DN44 (MPXV), P20509 (VACV), P0DOO5 (VARV)	DNA polymerase	GO:0003677	DNA binding	F	ECO:0000501
A0A7H0DN44 (MPXV), P20509 (VACV), P0DOO5 (VARV)	DNA polymerase	GO:0003824	catalytic activity	F	ECO:0007322
A0A7H0DN44 (MPXV), P20509 (VACV), P0DOO5 (VARV)	DNA polymerase	GO:0003887	DNA-directed DNA polymerase activity	F	ECO:0000501
A0A7H0DN44 (MPXV), P20509 (VACV), P0DOO5 (VARV)	DNA polymerase	GO:0016740	transferase activity	F	ECO:0007322

A0A7H0DN44 (MPXV), P20509 (VACV), P0DOO5 (VARV)	DNA polymerase	GO:0016779	nucleotidyltransferase activity	F	ECO:0007322
A0A7H0DN44 (MPXV), P20509 (VACV), P0DOO5 (VARV)	DNA polymerase	GO:0016787	hydrolase activity	F	ECO:0007322
P20509 (VACV), P0DOO5 (VARV)	DNA polymerase	GO:0034061	DNA polymerase activity	F	ECO:0007322
A0A7H0DN44 (MPXV), P20509 (VACV), P0DOO5 (VARV)	DNA polymerase	GO:0006260	DNA replication	P*	ECO:0007322
P0DOO5 (VARV)	DNA polymerase	GO:0006261	DNA-templated DNA replication	P	ECO:0007826
A0A7H0DN44 (MPXV), P20509 (VACV), P0DOO5 (VARV)	DNA polymerase	GO:0006310	DNA recombination	P	ECO:0007322
A0A7H0DN44 (MPXV), P20509 (VACV), P0DOO5 (VARV)	DNA polymerase	GO:0019079	viral genome replication	P	ECO:0000256
A0A7H0DN44 (MPXV), P20509 (VACV), P0DOO5 (VARV)	DNA polymerase	GO:0039693	viral DNA genome replication	P	ECO:0007322

*F: molecular function GO category, P: biological process GO category

Supplementary Table 4. 50 potential candidates with broad-spectrum inhibitory activity on the poxvirus DNA polymerases.

Name	CAS Number	Drug Status	SMILES	Formula	Drug Bank ID	PubChem CID	ChEMBL ID	DEEPScreen Confidence	Docking Score
Bis(Adenosine)-5'-Triphosphate		experimental	[H][C@]1(COP(O)(=O)OP(O)(=O)OP(O)(=O)OC[C@@]2([H])O[C@@]([H])(N3C=NC4=C(N)N=CN=C34)[C@]([H])(O)[C@]2([H])O)O[C@@]([H])(N2C=NC3=C(N)N=CN=C23)[C@]([H])(O)[C@]1([H])O	C20H27N10O16P3	DB01690	165381	CHEMBL407938	1.00000	-11.0794
Bictegravir	1611493-60-7	approved; investigational	OC1=C2N(C[C@H]3O[C@@H]4CC[C@@H](C4)N3C2=O)C=C(C(=O)NCC2=C(F)C=C(F)C=C2F)C1=O	C21H18F3N3O5	DB11799	90311989	CHEMBL3989866	1.00000	-10.4255
6-hydroxy-FAD	52301-43-6	experimental	CC1=CC2=C(N=C3C(=O)NC(=O)N=C3N2C[C@H](O)[C@H](O)[C@H](O)COP(O)(=O)OP(O)(=O)OC[C@H]2O[C@H]([C@H](O)[C@@H]2O)N2C=NC3=C2N=CN=C3N)C(O)=C1C	C27H33N9O16P2	DB02654	4369241		0.69444	-10.3187
7-methyl-GpppA		experimental	CN1C=[N+]([C@@H]2O[C@H](C	C21H30N10O17P3	DB01649	446336		0.80556	-10.2649

			O[P@](O)(=O)O[P@](O)(=O)O[P@](O)(=O)OC[C@H]3O[C@H]([C@H](O)[C@@H]3O)N3C=NC4=C(N)N=CN=C34)[C@@H](O)[C@H]2O)C2=C1C(=O)N=C(N)N2						
Bis(5-Amidino-2-Benzimidazolyl)Methane Ketone Hydrate		experimental	NC(=N)C1=CC2=C(C=C1)N=C(N2)C(O)(O)C1=NC2=C(N1)C=C(C=C2)C([NH3+])=[NH2+]	C17H18N8O2	DB04301	131704276		0.69444	-10.0557
Cefmetazole	56796-20-4	approved; investigational	[H][C@]12SCC(CSC3=NN=NN3C)=C(N1C(=O)[C@]2(NC(=O)CSCC#N)OC)C(O)=O	C15H17N7O5S3	DB00274	42008	CHEMBL1201195	0.88889	-9.68544
Rotigaptide	355151-12-1	investigational	C[C@@H](NC(=O)CNC(=O)[C@H]1C[C@H](O)CN1C(=O)[C@H]1CCCN1C(=O)[C@@H](CC1=CC=C(O)C=C1)NC(C)=O)C(=O)NCC(N)=O	C28H39N7O9	DB13067	9938933	CHEMBL450656	1.00000	-9.24707
[N-[N-(4-Methoxy-2,3,6-trimethylphenylsulfonyl)-L-aspartyl]-D-(4-amidino-phenylalanyl)]-piperidine	146663-95-8	experimental	COC1=C(C)C(C)=C(C(C)=C1)S(=O)(=O)N[C@@H](CC(O)=O)C(=O)N[C@H](CC1=CC=C(C=C1)C(N)=N)C(=O)N1CCCCC1	C29H39N5O7S	DB03081	177837	CHEMBL1231689	0.91667	-9.19588

Aeruginosin 98-B		experimental	[H]N([H])C(=NCCCCN([H])C(=O)[C@@H]1C[C@]2([H])CC[C@H](C[C@]2([H])N1C(=O)[C@H](N([H])C(=O)[C@H](O)CC1=CC=C(O)C=C1)[C@H](C)CC)OS(O)(=O)=O)N([H])[H]	C29H46N6O9S	DB04391	444346		1.00000	-8.93904
Cefminox	84305-41-9	experimental	[H][C@]12SCC(CSC3=NN=NN3C)=C(N1C(=O)[C@]2(NC(=O)CSC[C@@H](N)C(O)=O)OC)C(O)=O	C16H21N7O7S3	DB09062		CHEMBL1276342	0.75000	-8.91945
1-(2-{[(6-amino-2-methylpyridin-3-yl)methyl]amino}ethyl)-6-chloro-3-[(2,2-difluoro-2-pyridin-2-ylethyl)amino]-1,4-dihydropyrazin-2-ol		experimental	CC1N[C@@H](N)CCC1CNCCN1C(O)C(NCC(F)(F)C2=CC=CC=N2)=NC=C1Cl	C20H30ClF2N7O	DB07515	131704304		0.88889	-8.65452
1-GUANIDINO-4-(N-NITRO-BENZOYLAMINO-L-LEUCYL-L-PROLYLAMINO)BUTANE		experimental	CC(C)C[C@H](NC(=O)C1=CC=C(C=C1)[N+]([O-])=O)C(=O)N1CCC[C@H]1C(=O)NCCCCN=C(N)N	C23H35N7O5	DB04771	5494439		0.38889	-8.55984
Venglustat	1401090-53-6	investigational	CC(C)(NC(=O)O[C@@H]1CN2CCC1CC2)C1=CSC(=N1)C	C20H24FN3O2S	DB14966			0.36111	-8.41592

			1=CC=C(F)C=C1						
Infigratinib	872511-34-7	investigational	CCN1CCN(CC1)C1=CC=C(NC2=CC(=NC=N2)N(C)C(=O)NC2=C(Cl)C(OC)=CC(OC)=C2Cl)C=C1	C26H31Cl2N7O3	DB11886	53235510	CHEMBL1852688	1.00000	-8.25729
Dubermatinib	1341200-45-0	investigational	CN(C)S(=O)(=O)C1=CC=CC=C1NC1=NC(NC2=CC=C(CN3CCN(C)CC3)C=C2)=NC=C1Cl	C24H30ClN7O2S	DB15187		CHEMBL2022968	1.00000	-8.1971
1-(5-{2-[(1-methyl-1H-pyrazolo[4,3-d]pyrimidin-7-yl)amino]ethyl}-1,3-thiazol-2-yl)-3-[3-(trifluoromethyl)phenyl]urea		experimental	CN1N=CC2=C1C(NCCC1=CN=C(NC(=O)NC3=CC=CC(=C3)C(F)(F)F)S1)=NC=N2	C19H17F3N8OS	DB07362	15602701		0.44444	-7.98224
3-chloro-5-[2-chloro-5-(1H-pyrazolo[3,4-b]pyridin-3-ylmethoxy)phenoxy]benzonitrile		experimental	ClC1=CC(OC2=C(Cl)C=CC(OCC3=NNC4=NC=CC=C34)=C2)=CC(=C1)C#N	C20H12Cl2N4O2	DB08459	16045340	CHEMBL491019	0.63889	-7.73675
Afabicin	1518800-35-5	investigational	CN(CC1=C(C)C2=CC=CC=C2O1)C(=O)\C=C\C1=CN=C2N(COP(O)(O)=O)C(=O)CCC2=C1	C23H24N3O7P	DB15268			0.75000	-7.72848
JNJ-41443532	1228650-83-6	investigational	O[C@]1(CC[C@@H](CC1)N1CC(	C22H25F3N4O3S	DB12632	46208367	CHEMBL2204263	0.97222	-7.68658

			C1)NC(=O)CNC(=O)C1=CC=CC(=C1)C(F)(F)F)C1=CN=CS1						
Relenopride	1221416-43-8	investigational	COC1=CC(N)=C(Cl)C=C1C(=O)NC1CCN(CC[C@H](OC(N)=O)C2=CC=C(F)C=C2)CC1	C24H30ClFN4O4	DB12798	45275554	CHEMBL3544975	0.30556	-7.49579
Ciraparantag	1438492-26-2	investigational	N[C@@H](CCCNC(N)=N)C(=O)NCCN1CCN(CCCNC(=O)[C@@H](N)CCCNC(N)=N)CC1	C22H48N12O2	DB15199		CHEMBL3544919	0.22222	-7.48655
Itacitinib	1334298-90-6	investigational	FC1=C(N=CC=C1C(=O)N1CCC(CC1)N1CC(CC#N)(C1)N1C=C(C=N1)C1=C2C=CNC2=NC=N1)C(F)(F)F	C26H23F4N9O	DB12154	53380437	CHEMBL3622820	0.88889	-7.47209
Oteseconazole	1340593-59-0	investigational	O[C@@](CN1C=NN=N1)(C1=CC=C(F)C=C1F)C(F)(F)C1=CC=C(C=N1)C1=CC=C(OCC(F)(F)F)C=C1	C23H16F7N5O2	DB13055	77050711	CHEMBL3311228	0.66667	-7.38818
ABT-384	868623-40-9	investigational	CC(C)(N1CCN(CC1)C1=NC=C(C=C1)C(F)(F)F)C(=O)N[C@H]1C2CC3CC1C[C@@](C3)(C2)C(N)=O	C25H34F3N5O2	DB12501	11670435		0.97222	-7.29433
Cefditoren	104145-95-1	approved; investigational	[H][C@]12SCC(\C=C/C3=C(C)N=CS3)=C(N1C(=O)[C@H]2NC(=O)	C19H18N6O5S3	DB01066	9870843	CHEMBL1743	0.25000	-7.18414

			C(=N/OC)\C1=CSC(N)=N1)C(O)=O						
PCO-371	1613373-33-3	investigational	CC1=CC(=CC(C)=C1CCS(=O)(=O)N1CCC2(CC1)N=C(NC2=O)C1=CC=C(OC(F)(F)F)C=C1)N1C(=O)NC(=O)C1(C)C	C29H32F3N5O6S	DB14946		CHEMBL3976807	0.63889	-7.10586
4-[[(1E)-2-(4-CHLOROPHENYL)ETHENYL]SULFONYL]-1-[[1-(4-PYRIDINYL)-4-PIPERIDINYL]METHYL]PIPERAZINONE		experimental	ClC1=CC=C(\C=C\S(=O)(=O)N2CCN(CC3CCN(CC3)C3=CC=NC=C3)C(=O)C2)C=C1	C23H27ClN4O3S	DB08745	446345		1.00000	-7.05976
Etravirine	269055-15-4	approved	CC1=CC(=CC(C)=C1OC1=C(Br)C(N)=NC(NC2=CC=C(C=C2)C#N)=N1)C#N	C20H15BrN6O	DB06414	193962	CHEMBL308954	0.77778	-7.0398
Vilazodone	163521-12-8	approved	NC(=O)C1=CC2=CC(=CC=C2O1)N1CCN(CCCCC2=CNC3=C2C=C(C=C3)C#N)CC1	C26H27N5O2	DB06684	6918314	CHEMBL439849	1.00000	-6.97502
THIENO[3,2-B]PYRIDINE-2-SULFONIC ACID [1-(1-AMINO-ISOQUINOLIN-7-YLMETHYL)-2-OXO-PYRROLDI		experimental	[H][C@@]1(CCN(CC2=CC3=C(C=CN=C3N)C=C2)C1=O)NS(=O)(=O)C1=CC2=C(S1)C=CC=N2	C21H19N5O3S2	DB07261	445480	CHEMBL316053	0.50000	-6.9557

N-3-YL]-AMIDE									
(2S)-6-(2,4-DIAMINO-6-ETHYLPYRIMIDIN-5-YL)-2-(3,5-DIFLUOROPHENYL)-4-(3-METHOXYPROPYL)-2H-1,4-BENZOXAZIN-3(4H)-ONE		experimental	[H][C@]1(OC2=C(C=C(C=C2)C2=C(N)N=C(N)N=C2CC)N(CCCOC)C1=O)C1=CC(F)=CC(F)=C1	C24H25F2N5O3	DB07113	20843156		1.00000	-6.95552
JNJ-42165279	1346528-50-4	investigational	FC1(F)OC2=CC=C(CN3CCN(CC3)C(=O)NC3=C(Cl)C=CN=C3)C=C2O1	C18H17ClF2N4O3	DB15173			1.00000	-6.85018
Clazosentan	180384-56-9	investigational	COC1=CC=CC=C1OC1=C(NS(=O)(=O)C2=CC=C(C)C=N2)N=C(N=C1OCCO)C1=CC=NC(=C1)C1=NN=NN1	C25H23N9O6S	DB06677		CHEMBL109648	0.05556	-6.72949
[4-(6-Chloro-Naphthalene-2-Sulfonyl)-Piperazin-1-Yl]-(3,4,5,6-Tetrahydro-2h-[1,4']Bipyridinyl-4-Yl)-Methanone		experimental	ClC1=CC=C2C=C(C=CC2=C1)S(=O)(=O)N1CCN(CC1)C(=O)C1CCN(CC1)C1=CC=NC=C1	C25H27ClN4O3S	DB01836	446399	CHEMBL270527	0.11111	-6.71632
Elexacaftor	2216712-66-0	approved; investigational	C[C@@H]1CN(C2=NC(=CC=C2C(=O)NS(=O)(=O)C2=CN(C)N=C2C)N2C=CC(O	C26H34F3N7O4S	DB15444			0.30556	-6.59869

			CC(C)(C)C(F)(F)F)=N2)C(C)(C)C1						
Fagrocorat	1044535-52-5	experimental	[H][C@]12CCC3=C(C=CC(=C3)C(=O)NC3=C(C)N=CC=C3)[C@]1(CC1=CC=CC=C1)CC[C@@](O)(C2)C(F)(F)F	C29H29F3N2O2	DB14676		CHEMBL3137304	0.00000	-6.54472
Cefotetan	69712-56-7	approved	[H][C@]12SCC(CSC3=NN=NN3C)=C(N1C(=O)[C@]2(NC(=O)C1SC(S1)=C(C(N)=O)C(O)=O)OC)C(O)=O	C17H17N7O8S4	DB01330	53025	CHEMBL474579	0.77778	-6.47066
Rilpivirine	500287-72-9	approved	CC1=CC(\C=C\C#N)=CC(C)=C1NC1=CC=NC(NC2=CC=C(C=C2)C#N)=N1	C22H18N6	DB08864	6451164	CHEMBL175691	0.94444	-6.46011
7-((Carboxy(4-Hydroxyphenyl)Acetyl)Amino)-7-Methoxy-(3-((1-Methyl-1h-Tetrazol-5-Yl)Thio)Methyl)-8-Oxo-5-Oxa-1-Azabicyclo[4.2.0]Oct-2-Ene-2-Carboxylic Acid		experimental	CO[C@]1(NC(=O)[C@H](C(O)=O)C2=CC=C(O)C=C2)[C@H]2OCC(CSC3=NN=NN3C)=C(N2C1=O)C(O)=O	C20H20N6O9S	DB04342	5488645		0.97222	-6.44651
Cefmenoxime	65085-01-0	approved	[H][C@]12SCC(CSC3=NN=NN3C)=C(N1C(=O)[C@H]2NC(=O)C(=N/OC)\C1=C	C16H17N9O5S3	DB00267	9570757	CHEMBL1201224	1.00000	-6.38759

			SC(N)=N1)C(O)=O						
Relacorilant	1496510-51-0	investigational	CN1C=C(C=N1)S(=O)(=O)N1CCC2=CC3=C(C[C@@]2(C1)C(=O)C1=CC(=CC=N1)C(F)(F)F)C=NN3C1=CC=C(F)C=C1	C27H22F4N6O3S	DB14976		CHEMBL4068611	1.00000	-6.29723
1-[[(1E)-2-(4-CHLOROPHENYL)ETHENYL]SULFONYL]-4-[[1-(4-PYRIDINYL)-4-PIPERIDINYL]METHYL]PIPERAZINE		experimental	ClC1=CC=C(\C=C\S(=O)(=O)N2CCN(CC3CCN(CC3)C3=CC=NC=C3)CC2)C=C1	C23H29ClN4O2S	DB08746	446346		1.00000	-5.9628
2-CHLORO-5-(3-CHLORO-PHENYL)-6-[(4-CYANO-PHENYL)-(3-METHYL-3H-IMIDAZOL-4-YL)-METHOXYMETHYL]-NICOTINONITRILE		experimental	[H][C@](OCC1=NC(Cl)=C(C=C1C1=CC(Cl)=CC=C1)C#N)(C1=CN=CN1C)C1=CC=C(C=C1)C#N	C25H17Cl2N5O	DB06953	5287467		0.05556	-5.41473
TAS-116	1260533-36-5	investigational	CCC1=CC(=CC=C1N1N=C(C(C)C)C2=C1N=CC=C2N1C=NC(=C1)C1=CN(C)N=C1)C(N)=O	C25H26N8O	DB14876		CHEMBL3661115	0.88889	-5.3844
Tipifarnib	192185-72-1	investigational	CN1C=NC=C1[C@@](N)(C1=CC=C(Cl)C=C1)C1=CC2=C(	C27H22Cl2N4O	DB04960	159324	CHEMBL289228	1.00000	-5.38333

			C=C1)N(C)C(=O)C=C2C1=CC(Cl)=CC=C1						
ZK-806711		experimental	[H]N=C(C)N1CCC(CC1)OC1=CC2=C(C=C1)N=C(C)N2CC1=CC2=C(C=C1)C=CC(=C2)C(N)=[NH2+]	C27H31N6O	DB03373	131704242		0.91667	-5.2765
Fosdagrocorat	1044535-58-1	investigational	[H][C@]12CCC3=C(C=CC(=C3)C(=O)NC3=C(C)N=CC=C3)[C@]1(CC1=CC=CC=C1)CC[C@](C2)(OP(O)(O)=O)C(F)(F)F	C29H30F3N2O5P	DB12198	24872952	CHEMBL3137316	1.00000	-5.24937
1-{4-[4-Amino-6-(4-methoxyphenyl)furo[2,3-d]pyrimidin-5-yl]phenyl}-3-[2-fluoro-5-(trifluoromethyl)phenyl]urea		experimental	COC1=CC=C(C=C1)C1=C(C2=C(N)N=CN=C2O1)C1=CC=C(NC(=O)NC2=C(F)C=CC(=C2)C(F)(F)F)C=C1	C27H19F4N5O3	DB04727	5326956	CHEMBL194911	0.97222	-4.83856
N-(3-cyanophenyl)-2'-methyl-5'-(5-methyl-1,3,4-oxadiazol-2-yl)-4-biphenylcarboxamide		experimental	CC1=NN=C(O1)C1=CC=C(C)C(=C1)C1=CC=C(C=C1)C(=O)NC1=CC(=CC=C1)C#N	C24H18N4O2	DB07833	10200651	CHEMBL270164	0.75000	-4.6905
6-[3-(4-Morpholinyl)Propyl]-2-(3-Nitrophenyl)-5-Thioxo-5,6,-		experimental	[O-][N+](=O)C1=CC(=CC=C1)C1=CC2=C(S1)C=C1N2C(=S)N(CCCN2CC	C21H20N4O4S2	DB03507	4470566		0.11111	-4.39668

Dihydro-7h-Thienol[2',3':4,5]Pyrrolo[1,2-C]Imidazol-7-One			OCC2)C1=O						

Supplementary Table 5. Key resources used in this study

REAGENT or RESOURCE	SOURCE	IDENTIFIER
Antibodies		
Anti-double-stranded-RNA antibody (SCIONS J2 monoclonal antibody)	English&Scientific Consulting Kft	Cat# 10010200
Rabbit polyclonal anti-Vaccinia virus Lister Strain (FITC)	Abbexa	Cat# abx023199
Anti-EpCAM antibody	Abcam	Cat# ab71916
Goat anti-Mouse IgG (H+L) Highly Cross-Adsorbed Secondary Antibody (Alexa Fluor Plus 594)	Thermo Fisher Scientific	Cat# A32742
Anti-rabbit IgG (H+L), F(ab')2 Fragment (Alexa Fluor®488 Conjugate)	Bioké	Cat# 4412S
Vectashield with DAPI	Thermo Fisher Scientific	Cat# 13285184
Anti-Cytokeratin 7 antibody	abcam	Cat# ab9021
Virus strains		
Monkeypox virus clade IIb	European Virus Archive	Ref-SKU:010V-04721
Monkeypox virus clade Ia	Robert Koch Institute, Germany	
Monkeypox virus clade Ib	European Virus Archive	Ref-SKU: 012V-06039
VACV Elstree virus	Erasmus Medical Center, Netherlands	pmid:15919938
Cowpox virus strain HumGri07/1	European Virus Archive Global	Ref-SKU: 010V-01806
Chemicals		

CONTINUED		
REAGENT or RESOURCE	SOURCE	IDENTIFIER
Bictegravir	MedChemExpress	Cat# HY-17605
Etravirine	MedChemExpress	Cat#HY-90005
Cefmetazole sodium	MedChemExpress	Cat# HY-B1257
Cefditoren sodium	MedChemExpress	Cat#HY-17452
Dimethyl Sulfoxide for Synthesis (DMSO)	Sigma-Aldrich Chemie BV	Cat# D2650
MagNA Pure 96 External Lysis Buffer	Roche Diagnostics Nederland BV	Cat# 6374913001
Y27632	Bio-Techne	Cat# 1254/10
SB202190	Bio-Techne	Cat# 1264/10
FGF10	Peprotech	Cat# AF-100-26-1000
FGF7	Tebu-Bio BV	Cat# 100-19
Nicotinamide	Sigma-Aldrich Chemie BV	Cat# N0636
N-acetylcysteine	Sigma-Aldrich Chemie BV	Cat# A9165
B27	Thermo Fisher Scientific	Cat# 12587001
DMEM high glucose w/Na pyruvate w/ Stable glutamine	VWR International BV	Cat# L0193-500
Advanced DMEM/F12	Life Technologies Europe BV	Cat# 12634028
UltraGlutamine I (Alanyl-L-Glutamine) 200 mM (100X)	Westburg	Cat# LO BE17-605E/U1
Hepes	BioWhittaker	Cat# 17-737E
GlutaMAX™ Supplement	Gibco	Cat# 35050-038
PenicillinStreptomycin Solution, liquid	Life Technologies Europe BV	Cat# 11548876
DPBS, no calcium, no	Gibco	Cat#14190250

magnesium		
Fetal calf serum Continued	Gibco	Cat#10270
CONTINUED		
REAGENT or RESOURCE	SOURCE	IDENTIFIER
Cultrex PathClear Reduced Growth Factor BME	Bio-Techne	Cat# 3533-010-02
Critical commercial assays		
SYBR Select Master Mix for CFX-10 x	Thermo Fisher Scientific	Cat# 4472954
Macherey-Nagel<br>NucleoSpin DNA Kit	Bioke	740952.250
Oligonucleotides<br>(Primers)		
GGCTCTTCTATCAACCACA	This manuscript	MPXV
AGTCATTATCTCCTCCTCC<br>A	This manuscript	MPXV
CATCATCTGGAATTGTCACTACTAAA	This manuscript	poxvirus
ACGGCCGACAATATAATTAATGC	This manuscript	poxvirus
Software		
Graphpad Prism 8	GraphPad<br>software	https://www.graphpad.com/
Mega 7	Mega software	https://www.megasoftware.net/
Pymol	Pymol software	https://learn.schrodinger.com/public/pymol/20234/Content/pymol/pymol_home.htm

References

1. Jiang, S. *et al.* Generic Diagramming Platform (GDP): a comprehensive database of high-quality biomedical graphics. *Nucleic Acids Research* **53**, D1670-D1676 (2024). https://doi.org/10.1093/nar/gkae973
