# Supplementary material for: AI-driven discovery of antiretroviral drug bictegravir and etravirine as inhibitors against monkeypox and related poxviruses
Source: Commun Biol. 2025 Dec 2;8:1734. doi: 10.1038/s42003-025-09129-x (PMC12673143; doi:10.1038/s42003-025-09129-x)
Supplement: Supplementary file 2 — Description of Additional Supplementary Materials [file 42003_2025_9129_MOESM2_ESM.pdf]

Description of Additional Supplementary Files

File name: Supplementary Data 1-5

Description: The source data behind the graphs in the paper
